# Synthesis of Thiomorpholine via a Telescoped Photochemical Thiol–Ene/Cyclization Sequence in Continuous Flow

**DOI:** 10.1021/acs.oprd.2c00214

**Published:** 2022-08-04

**Authors:** Alexander Steiner, Ryan C. Nelson, Doris Dallinger, C. Oliver Kappe

**Affiliations:** † Institute of Chemistry, University of Graz, NAWI Graz, Heinrichstrasse 28, 8010 Graz, Austria; ‡ Center for Continuous Flow Synthesis and Processing (CCFLOW), Research Center Pharmaceutical Engineering GmbH (RCPE), Inffeldgasse 13, 8010 Graz, Austria; § Medicines for All Institute, Virginia Commonwealth University, 737 North Fifth Street, P.O. Box 980100, Richmond, Virginia 23298, United States

**Keywords:** thiol−ene reaction, thiomorpholine, vinyl chloride, photochemistry, continuous flow

## Abstract

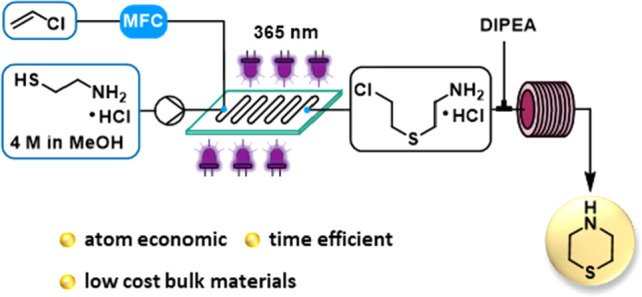

A procedure for the continuous flow generation of thiomorpholine in a two-step telescoped format was developed. The key step was the photochemical thiol–ene reaction of cysteamine hydrochloride and vinyl chloride as low-cost starting materials. This reaction could be conducted under highly concentrated (4 M) conditions using a low amount (0.1–0.5 mol %) of 9-fluorenone as the photocatalyst, leading to the corresponding half-mustard intermediate in quantitative yield. Thiomorpholine was subsequently obtained by base-mediated cyclization. The robustness of the process was demonstrated by performing the reaction for 7 h (40 min overall residence time), isolating the desired thiomorpholine via distillation.

## Introduction

**Scheme 1:**
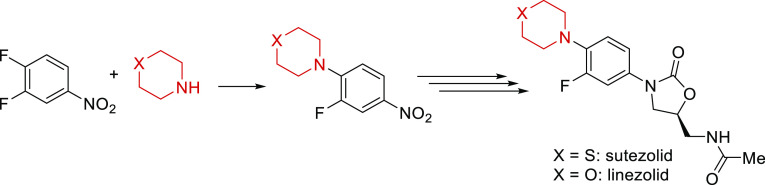
First Reaction Step Toward Sutezolid/Linezolid

The thiomorpholine moiety is an important structural motif that is incorporated into a variety of active pharmaceutical ingredients because of its interesting pharmacological profile, including antimalarial, antibiotic, antioxidant, or hypolipidemic activity.^1^ A prominent example is the oxazolidinone antibiotic sutezolid that is currently in phase 2 clinical trials for the treatment of multidrug-resistant tuberculosis. Due to its improved therapeutic potential, it is considered to be a promising replacement for the FDA-approved, first-generation drug linezolid, the morpholine analog of sutezolid (Scheme 1).^2^ However, the Medicines for All Institute conducted techno-economic analyses of the routes toward sutezolid (see Scheme S1)^3,4^ that identified thiomorpholine as the most significant cost driver. In order to be cost competitive with linezolid and therefore accessible to low- and middle-income countries, a scalable route to generate thiomorpholine from low-cost starting materials is highly desirable.

**Scheme 2:**
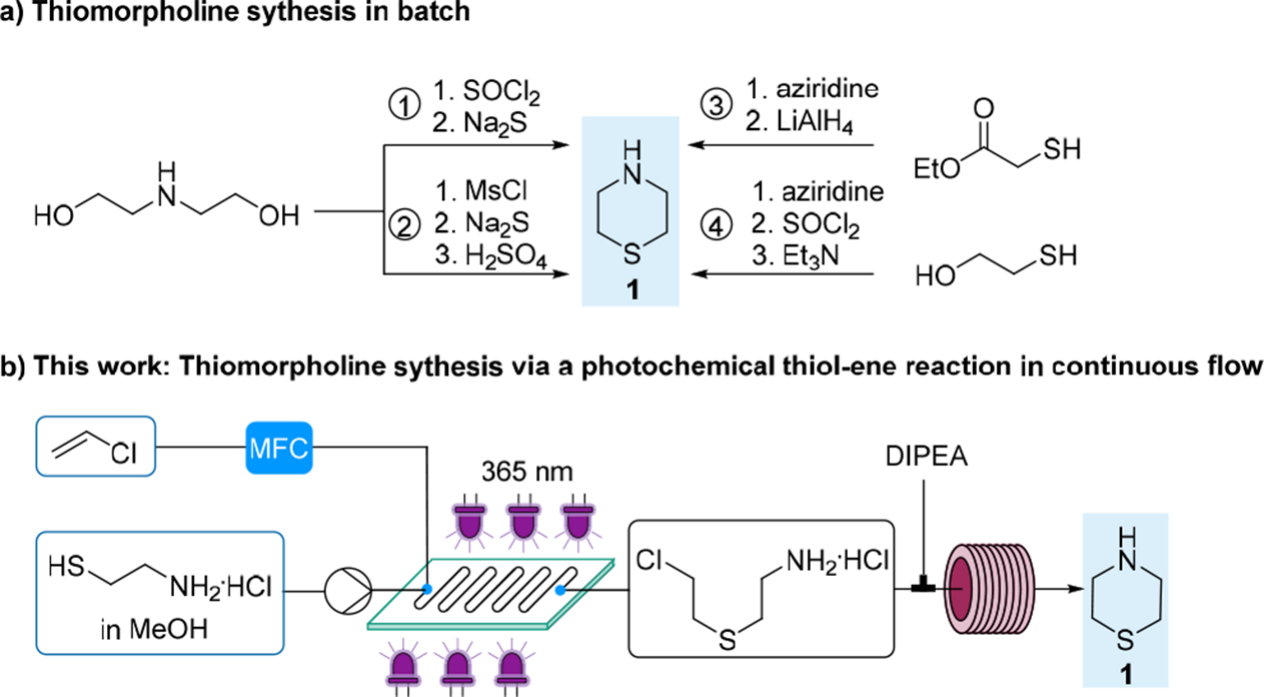
Syntheses toward Thiomorpholine

Approaches toward the synthesis of thiomorpholine (**1**) are displayed in Scheme 2a and include the transformation of diethanolamine into **1** via generation of an amino-mustard species and its cyclization by treatment with sodium sulfide (routes 1 and 2).^5,6^ Starting from ethyl mercaptoacetate and aziridine, **1** can be obtained by LiAlH_4_ reduction of the generated thiomorpholin-3-one (route 3).^7^ Another strategy involves the reaction of 2-mercaptoethanol with aziridine and further conversion to 2-(2-chloroethylthio)ethylamine hydrochloride, which is then cyclized with Et_3_N to **1** (route 4).^8^ These procedures are rather time consuming (2–54 h), and isolation of thiomorpholine is achieved in 44–81% overall yield after either a distillative work-up^6−8^ or crystallization as the HCl salt.^5^ Although most of the reported routes use low-cost starting materials, they also generate nitrogen- or half-mustards, respectively, and thus producing these molecules on scale in a standard laboratory environment would be a safety challenge.

We were inspired to develop an alternative, time- and atom-efficient continuous flow route toward thiomorpholine, based on the thiol–ene reaction of cysteamine and vinyl chloride (VC). Thiol–ene reactions fall into the category of click chemistry due to their high yield, solvent and oxygen tolerance, and absence of byproducts.^9−11^ They proceed via a free-radical mechanism, and initiation is typically achieved either with UV irradiation or by thermolysis of a chemical additive. This strategy would lead to the same half-mustard 2-(2-chloroethylthio)ethylamine hydrochloride intermediate reported by Asinger et al. (route 4, Scheme 2a)^8^ in one step, which is then further cyclized without isolation under basic conditions to **1** (Scheme 2b). Both reagents are considered as low-cost bulk materials, with cysteamine itself being a high-volume FDA-approved drug, ensuring a stable supply. VC on the other hand, is one of the world’s most important commodity chemicals with an annual production of ca. 13 million metric tons.

However, since VC is supplied as a compressed, liquefied gas, highly toxic, flammable, and a Group 1 human carcinogen,^12^ and the generated intermediate is a half-mustard, the process is best conducted in a telescoped continuous flow format.^13,14^ Low reactor volumes ensure that only a small amount of material is present at any given time, and thus, hazardous materials can be safely handled in continuous flow.^15−18^ In addition, head space issues are eliminated, gaseous reagents can be dosed precisely, and processes can be seamlessly translated from the laboratory to industrial scale.^19,20^ Photochemical reactions typically can be improved when performed within the narrow channels of a microreactor, combined with state-of-the-art light-emitting diode (LED) irradiation technology.^21−24^

## Results and Discussion

### Batch Thiol–Ene Reactions

**Scheme 3:**
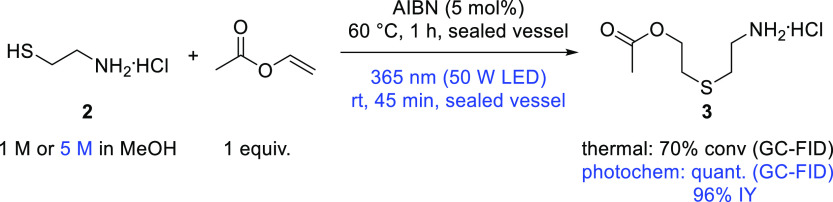
Thiol–Ene Reaction of Cysteamine Hydrochloride with Vinyl Acetate in Batch

Preliminary studies on the thermal and photochemical thiol–ene reaction were performed employing vinyl acetate as a more convenient and readily available alkene precursor on the laboratory scale, compared with VC. Methanol proved to be the solvent of choice, as cysteamine showed poor solubility in other solvents (e.g., MeCN, tetrahydrofuran, toluene, and DCM). These studies revealed that when cysteamine was used as a free base, the expected 2-aminoethylthioethyl acetate intermediate was not formed, but 2-methyl-1,3-thiazolidine was observed as the major product (Figures S1 and S2). Notably, when switching to cysteamine hydrochloride (**2**) under otherwise identical conditions, the desired product **3** was obtained (Scheme 3 and Figures S3–S5). The photochemical route not only provided full conversion but also a very clean reaction profile compared to the thermal reaction (Figure S6). Concentrations of up to 5 M of **2** in MeOH could be reached, and the photochemical protocol furnished **3** in 96% yield after a simple evaporation work-up (Scheme 3).

With this information in hand, we next turned our attention to VC, which is expected to be the superior alkene building block with respect to atom-economy, material availability, and reactivity.

**Scheme 4:**
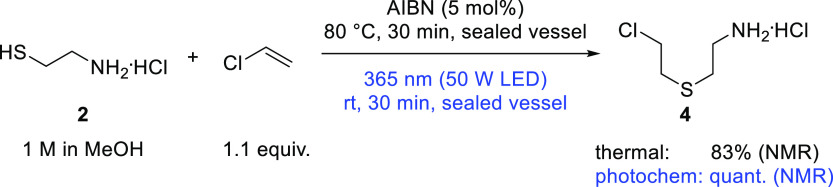
Thiol–Ene Reaction of Cysteamine Hydrochloride with VC in Batch

For batch optimizations, 1.1 equiv VC (bp −13.4 °C) was condensed into a 1 M solution of cysteamine hydrochloride (**2**) in MeOH (Figure S10). This procedure allowed more accurate dosing of VC compared to simply sparging the solution with VC, which results in a higher amount of VC in the headspace and thus less conversion to the product. A quick comparison of the thermal (80 °C, 30 min, 5 mol % AIBN) and photochemical (rt, 30–60 min, 365 nm) reaction provided similar results as those with vinyl acetate: the photochemical route proved to be highly selective, and a quantitative yield of 2-(2-chloroethylthio)ethylamine hydrochloride (**4**) by nuclear magnetic resonance (NMR) was obtained, versus 83% thermally (Scheme 4).

### Continuous Flow Photochemical Thiol–Ene Reaction

We moved forward to optimization studies in continuous flow using a commercial plate-based flow photoreactor (Corning AFR, Lab Photo Reactor).^25^ Since we encountered clogging issues during the thiol–ene reaction of **2** and vinyl acetate and were unsuccessful in the cyclization of **3** to thiomorpholine,^26^ no further optimization studies were performed (see Supporting Information and Figure S8).

For the thiol–ene reaction of **2** and VC, the set-up shown in Table 1 was used. VC was fed via a calibrated mass flow controller (MFC), which allowed accurate dosing of the gas. Since VC is provided as a compressed, liquefied gas at 3 bar, the maximum outlet pressure was limited to 1 bar, which prevented performing the reaction at gas flow rates higher than ca 12 mLn/min and thus higher throughput. In addition, the integration of a back pressure regulator (BPR) was not suitable, most likely resulting in less VC dissolved in solution and a lower residence time than calculated. Employing a 1 M solution of **2** in MeOH, a calculated maximum residence time^27^ of 10 min and irradiation at 365 nm, provided NMR yields of only 53–58% (Table 1, entries 1 and 2). By lowering the temperature from 20 to 6 °C, which was beneficial in the reaction with vinyl acetate, the yield dropped to 18% (entry 3).

**Table 1:**
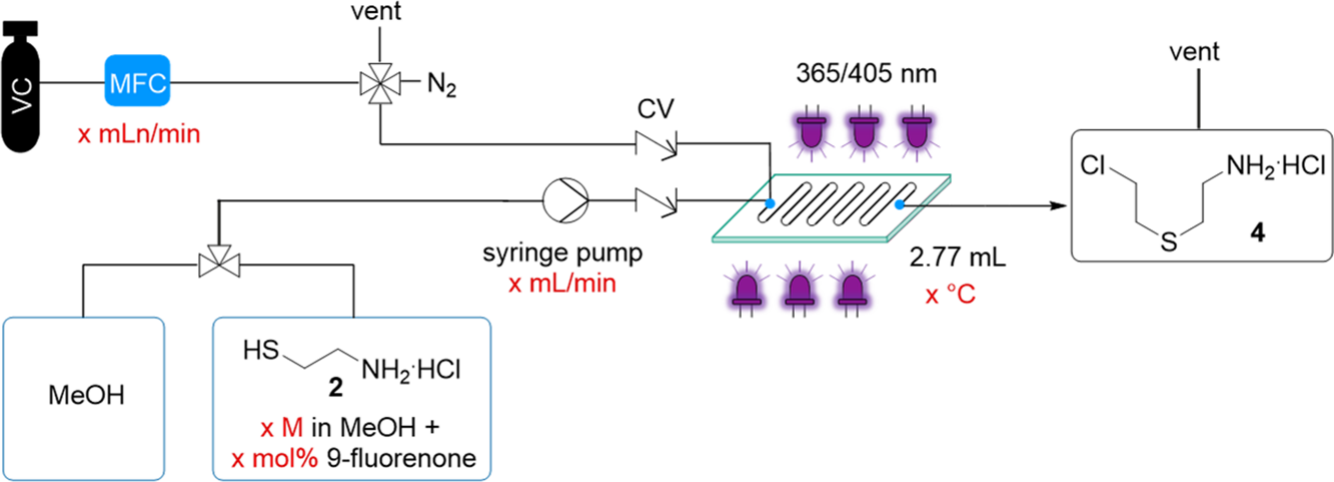
Optimization Studies of the Photochemical Thiol–Ene Reaction of 2 with VC in Continuous Flow^a^

						flow rates (mL/min)		
entry	conc of 2 (M)	9-FL (mol %)	*T* (°C)	equiv VC	wavelength (nm)	2	VC	NMR yield (%)
1	1		20	1.1	365	0.277	6.6	53
2	1		20	1.0	365	0.277	6.1	58
3	1		6	1.0	365	0.277	6.1	18
4^b^	1	5	20	1.0	365	0.277	6.1	87
5^b^	2	5	20	1.0	365	0.277	12.1	93
6^c^	4	5	20	1.0	365	0.139	12.1	>99
7	4	1	20	1.0	365	0.139	12.1	>99
**8**	**4**	**0.1**	**20**	**1.0**	**365**	**0.139**	**12.1**	**>99**
9	4		20	1.0	365	0.139	12.1	98
10	4	1	20	1.0	405	0.139	12.1	>99
11	4		20	1.0	405	0.139	12.1	54

a Conditions: liquid feed: **2**, 9-FL, and methyl benzoate as the internal standard were dissolved in MeOH (25 mL volumetric flask). CV: check valve. See the Experimental Section for more details and Table S1 for a full optimization table.

b Degassing of the liquid substrate feed with Ar.

c With or without degassing.

**Figure 1:**
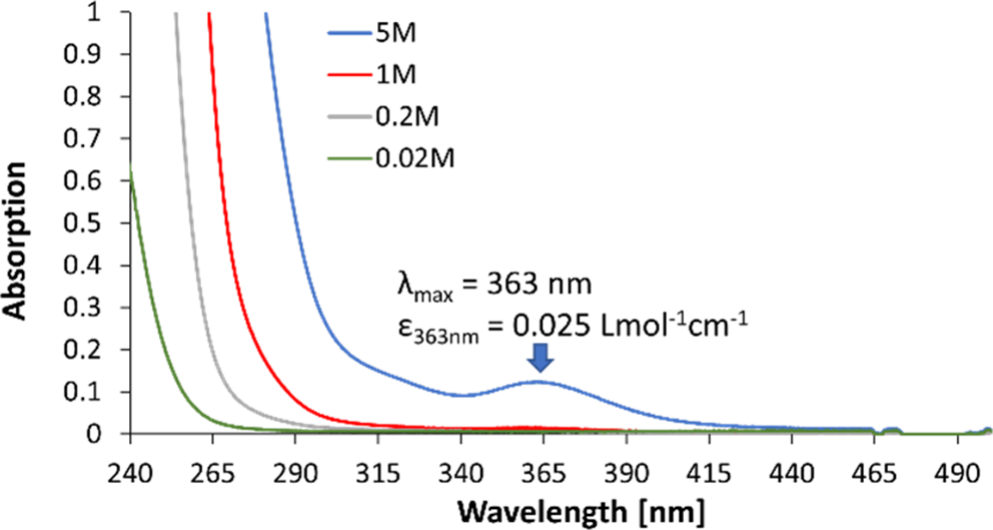
Absorption spectra of **2** at different concentrations in MeOH.

The poorer performance in flow was not unexpected since an extremely low absorption coefficient of **2** was determined (ε = 0.025 Lmol^–1^ cm^–1^ at 363 nm, Figure 1), resulting in an absorption of only about 1% of the incident light at a concentration of 1 M due to the short path length of 0.04 cm (plate’s channel size). Therefore, it appeared advantageous to employ a photocatalyst, a common practice for this photochemical reaction. For thiol–ene reactions in continuous flow, 2,2-dimethoxy-2-phenylacetophenone (DMPA) as a photoinitiator or Ru(bpy)_3_(PF_6_)_2_ as a photocatalyst have been reported.^28−30^ While Ru(bpz)_3_(PF_6_)_2_ has been reported to catalyze the thiol–ene reaction of similar thiol substrates such as cysteine methyl ester via a single electron transfer mechanism,^31^ it was important to avoid expensive metal catalysts in our approach. Therefore, to accelerate this reaction, we envisioned employing 9-fluorenone (9-FL) as an inexpensive photocatalyst.^32^ Although the oxidation potential of the T^1^ exited state of 9-FL (+0.96 V versus SCE)^33^ would suffice to oxidize cysteamine (+0.92 V versus SCE),^34^ it might also act via energy transfer catalysis.^35−37^ However, it is difficult to distinguish the two pathways experimentally.

As expected, the addition of 5 mol % of 9-FL increased the yield to 87% (entry 4). A further improvement in yield could only be accomplished by increasing the concentration of the liquid substrate feed (entries 5 and 6, see also Figure S9). The maximum achievable concentration was 4 M, providing **4** in quantitative yield. It has to be noted that for such highly concentrated solutions of **2**, dissolution is aided by sonication and that crystallization occurs upon cooling below room temperature. Typically, the flow rate of the substrate feed was kept constant (0.277 mL/min), and the flow rate of VC was adjusted accordingly. However, at a concentration of 4 M, the substrate feed flow rate had to be reduced to 0.139 mL/min because the MFC was unable to consistently deliver VC at a rate of 24.2 mLn/min (increased pressure downstream due to high flow rate). In turn, this increased the residence time to 20 min.

Although the thiol–ene reaction is rather insensitive toward O_2_, the photocatalyst might be quenched by O_2_. Hence, the liquid substrate feed containing **2**, methyl benzoate as the internal standard, and 9-FL was additionally degassed by sparging with argon for ca. 1 min. Interestingly, no change in reactivity was observed whether the liquid feed was degassed or not (entry 6). Further optimizations revealed that the sensitizer concentration can be reduced to 0.1 mol % (720 mg/L) without compromising the yield (entries 7 and 8). Remarkably, at such a high substrate concentration, the reaction proceeded to 98% yield (vs 58% at 1 M) even without a sensitizer (entry 9). Furthermore, 9-FL can also be excited at longer wavelengths. Irradiation at 405 nm yielded quantitative conversion when employing 1 mol % of 9-FL, while without the photocatalyst, the yield drops to 54% (entry 10 versus 11). Under optimized conditions—4 M solution of **2**, 1 equiv. VC, 0.1–5 mol % 9-FL, 365 nm, 20 min residence time—intermediate **4** was generated with a throughput of 5.9 g/h.

Interestingly, gas formation was observed at the reactor outlet, although only an equimolar amount of VC was employed. Therefore, an isolation experiment was performed. As the isolated yield was in agreement with the ^1^H NMR yield (see Supporting Information and Figures S12 and S13), no further investigations were conducted. It also has to be noted that over the course of the reaction, the volume of the liquid stream increased by 17%, resulting in a reduced concentration of intermediate **4** of 3.42 M.

### Telescoped Sequence toward Thiomorpholine

For the cyclization of intermediate **4** to thiomorpholine, a base screen was first performed in batch (Table S2). Full conversion of **4** was achieved, and thiomorpholine was obtained with an NMR yield of 86–89% after 5 min at 100 °C by employing 2 equiv of either Et_3_N, DIPEA, or DBU. Et_3_N was successfully used for this reaction by Asinger et al.;^8^ however, since precipitation was observed, this base was unsuitable for a flow protocol. With respect to cost efficiency, we decided to explore the telescoped thiol–ene/cyclization reaction with DIPEA.

**Scheme 5:**
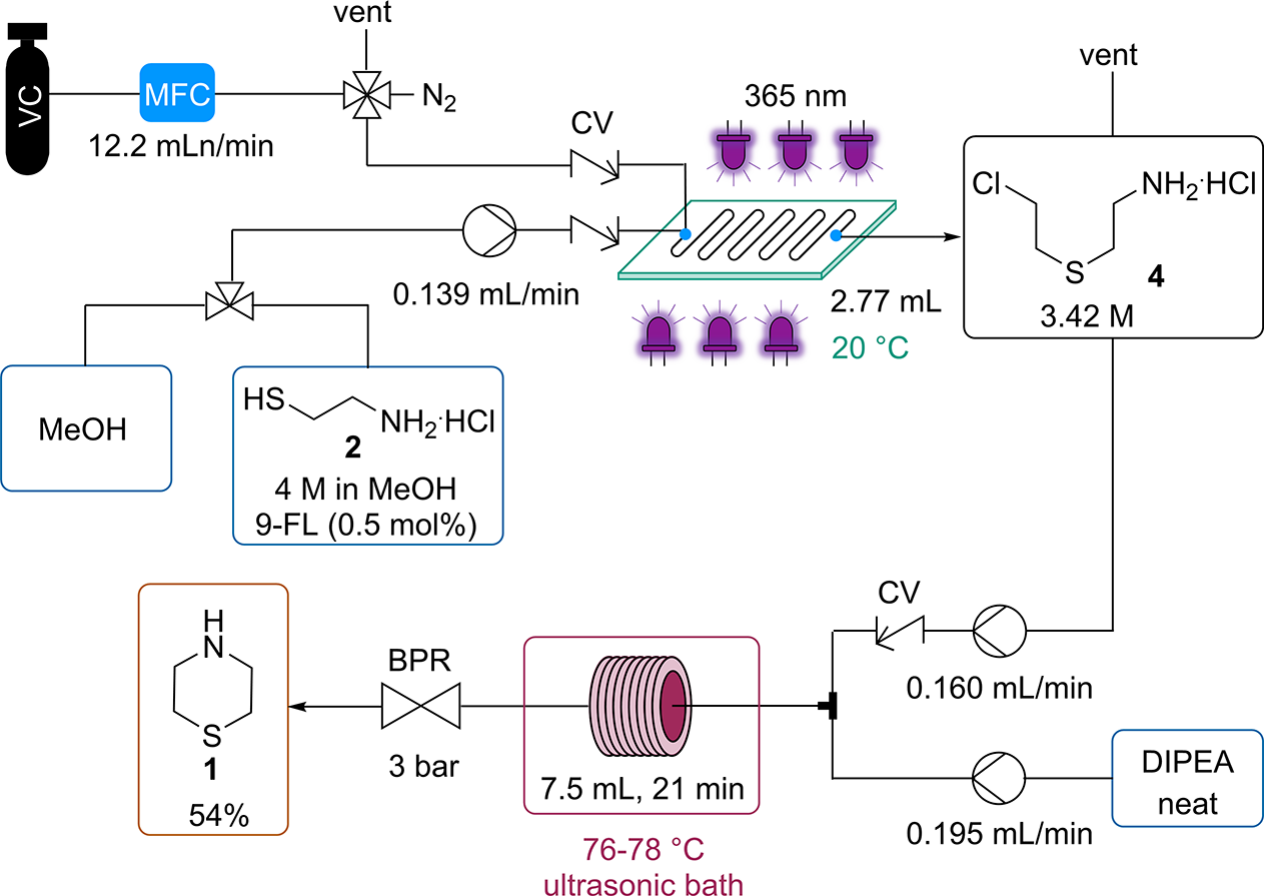
Telescoped Continuous Flow Procedure toward Thiomorpholine

Since the cyclization proceeds faster at temperatures above the boiling point of MeOH, a BPR set to 3 bar was installed. Due to an outlet pressure limitation of 1 bar of the VC cylinder, a hold vessel needed to be introduced to collect the exit stream of the thiol–ene reaction, which was then further pumped to be mixed with 2 equiv of neat DIPEA (Scheme 5 and Figure S16B). This hold vessel also functioned as a gas separator. The thiol–ene reaction was performed under the conditions depicted in entry 8 in Table 1, using 0.1 mol % of 9-FL. We realized that a simple T-mixer did not provide efficient mixing, resulting in lower thiomorpholine yields. When introducing a coil filled with glass beads (PFA, 1.6 mm ID, 3.2 mm OD, 0.5 mL void volume when filled) after the T-mixer, which functions as both a mixing and reaction unit, the same outcome as in batch was observed: full conversion of **4,** and an 87% NMR yield of thiomorpholine could be achieved at 100 °C and 5 min residence time.

**Figure 2:**
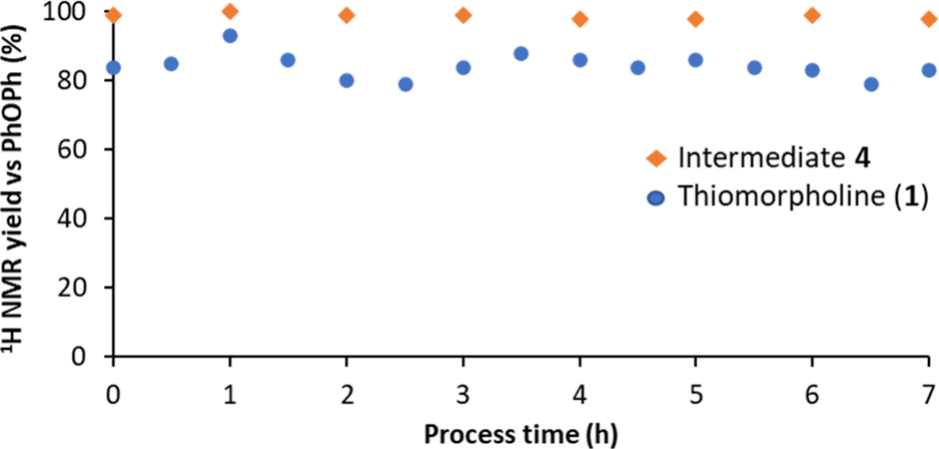
7 h run of the telescoped thiol–ene/cyclization sequence toward thiomorpholine. For conditions, see Scheme 5 and the Experimental Section.

Finally, we performed a long run to demonstrate the robustness of this process. For this purpose, the concentration of 9-FL was increased to 0.5 mol % to ensure a stable performance over a multi-hour run. After experiencing clogging issues with the above-mentioned setup at process times >1 h, a 7.5 mL coil (0.8 mm ID, 1.6 mm OD) that was immersed in an ultrasonic bath^38^ was used as the residence time unit at a temperature of 76–78 °C (Figure S16A). With this setup, the process was constant for 7 h after reaching a steady state (Scheme 5 and Figure 2). Yields of intermediate **4** and thiomorpholine of ≥98 and 84%, respectively, by NMR were achieved (Figure 2), which matches well with previous optimizations. After distillation, 12.74 g (54% overall) of thiomorpholine was isolated, which corresponds to a throughput of 1.8 g/h. The difference between isolated and NMR yield is related to losses during distillation (see the Supporting Information), which has not been fully optimized at this small scale. However, we expect these losses to be minimal when an improved work-up procedure is employed on a larger scale.

## Conclusions

In conclusion, we have developed a continuous flow process for the atom- and time-efficient generation of thiomorpholine by using readily available cysteamine (as its hydrochloride salt) and VC as bulk materials. The telescoped photochemical thiol–ene/cyclization sequence furnished thiomorpholine at the laboratory scale in 54% overall isolated yield (84% NMR yield) after distillation, which was comparable to most of the reported procedures (44–81%).^5−8^ In addition, compared to the routes described in the literature, which are potentially the most interesting in terms of production (routes 2 and 4, Scheme 2a), our route proved to be ca. 7 times more cost efficient.^39^

Key to this telescoped sequence was the continuous photochemical thiol–ene reaction, which proceeded under highly concentrated conditions (4 M solution of **2**), low amounts of 9-FL as the photocatalyst (≤0.5 mol %), and quantitative yield of **4**. Due to the low pressure of the VC gas cylinder, the throughput on the laboratory scale was limited to a maximum of 5.9 g/h for intermediate **4** and in turn of 1.8 g/h for thiomorpholine. To achieve higher production capacity, VC is best processed in the liquid form, as is common in polyvinyl chloride (PVC) production. On the laboratory scale, this technique is too high risk and impractical; however, with the appropriate equipment, this reaction has the potential to be safely scaled in a continuous flow format at the production scale. Several photochemical reactions have been demonstrated in continuous flow on production scales,^23,40−42^ which supports this assessment.

## Experimental Section

### General Remarks

All materials were purchased from commercial sources (TCI, Sigma-Aldrich, AirLiquide) and used without further purification. ^1^H NMR spectra were recorded on a Bruker 300 MHz instrument. ^13^C NMR spectra were recorded on the same instrument at 75 MHz. Chemical shifts (δ) are expressed in ppm, downfield from TMS as the internal standard. The letters s, d, t, q, m, and brs are used to indicate singlet, doublet, triplet, quadruplet, multiplet, and broad singlet. Gas chromatography (GC)–flame ionization detector (FID) chromatography was performed using a Shimadzu GC FID 230 gas chromatograph with a FID. Helium, used as the carrier gas (40 cm s^–1^ linear velocity), goes through a RTX-5MS column (30 m × 0.25 mm ID × 0.25 μm). The injector temperature is set to 280 °C. After 1 min at 50 °C, the column temperature is increased by 25 °C min^–1^ to 300 °C and then held for 4 min at 300 °C. The gases used in the detector for flame ionization are hydrogen and synthetic air (5.0 quality). GC–mass spectrometry (MS) analysis was performed on a Shimadzu GCMS-QP2010 SE coupled with a DSQ II (EI, 70 eV). A fused silica capillary column Rtx-5MS column (5% diphenyl, 95% dimethylpolysiloxane, 30 m × 0.25 mm × 0.25 μm) was used. The injector temperature was set at 280 °C. After 1 min at 50 °C, the oven temperature was increased by 25 °C/min to 300 °C and maintained at 300 °C for 3 min. As a carrier gas, helium at 40 cm s^–1^ linear velocity was used. MS conditions were ionization voltage of 70 eV and the acquisition mass range of 50–450 *m*/*z*. Mass spectral libraries (Wiley Registry of Mass Spectral Data 11th Edition, NIST/EPA/NIH Mass Spectral Library 14) were searched with NIST MS Search software. Liquid chromatography (LC)–MS analysis was carried out on a Shimadzu instrument using a C18 reversed-phase analytical column (150 × 4.6 mm, particle size 5 μm) using mobile phases A (H_2_O/MeCN 90:10 (v/v) + 0.1% HCOOH) and B (MeCN + 0.1% HCOOH) at a flow rate of 0.6 mL/min. The following gradient was applied: hold at 5% solvent B until 2 min, increase to 20% solvent B until 8 min, increase to 100% solvent B until 16 min, and hold at 100% solvent B until 22 min. Low resolution mass spectra were obtained on a Shimadzu LCMS-QP2020 instrument using electrospray ionization (ESI) in positive or negative mode. High resolution MS (HR-MS) measurements were performed using a Q-Exactive Hybrid Quadrupole-Orbitrap mass spectrometer following flow injection analysis of the re-dissolved sample with the Dionex Ultimate 3000 series high-performance liquid chromatography (HPLC)-system (Thermo Fisher Sci., Erlangen, Germany). The injection volume was 5 μL, and the flow was 200 μL min^–1^ of acetonitrile (>99.9% HPLC-grade; Chem-Lab NV, Zedelgem, Germany). The high-resolution mass spectrometer was fitted with a HESI-II atmospheric pressure ESI source. Nitrogen was used as the nebulizer and drying gas. ESI-MS measurements were performed in positive ionization mode using the following settings: spray voltage of 3.5 kV, capillary temperature of 250 °C, sheath gas flow rate of 5 instrument units (IU), auxiliary gas temperature of 50 °C, auxiliary gas flow rate of 3 IU, automatic gain control target of 1*e*,^6^ maximum injection time of 30 ms, and resolution of 140,000 (full width at half-maximum). High resolution mass spectra were extracted from a scanned mass range of *m*/*z* 100–300. Melting points were obtained on a Stuart melting point apparatus in open capillary tubes. Batch reactions above the boiling point of MeOH were performed in an Initiator+ single-mode microwave reactor from Biotage, using 2.5 mL Pyrex vials. The reaction temperature was controlled by an external infrared sensor. Reaction times refer to hold times at the temperature indicated. UV/vis spectra were recorded using a fiber-coupled Avantes Starline AvaSpec-2048 spectrometer and were processed using Avasoft 8.7 software. A commercial continuous flow photoreactor (Corning Advanced-Flow Lab Photo Reactor) was used.

### Caution

VC is a highly toxic, flammable, and carcinogenic gas. Laboratory personnel working with VC must familiarize themselves with the potential hazards and prevention measures. It is recommended to use a dedicated gas detector.

### Batch Procedure for the Photochemical Thiol–Ene Reaction of Cysteamine Hydrochloride with Vinyl Acetate

A 25 mL pear-shaped flask was charged with cysteamine hydrochloride (2.84 g, 25 mmol) and MeOH (5 mL). Dissolution was aided by sonication. Then, vinyl acetate (2.53 mL, 27.5 mmol, 1.1 equiv.) was added. The flask was closed, and the solution was irradiated for 1 h using a 365 nm, 50 W LED. After evaporation of the solvent and the remaining vinyl acetate under reduced pressure, 2-aminoethylthioethyl acetate **3** was obtained as an off-white solid (4.8 g, 96%). Mp: 60–64 °C; ^1^H NMR (300 MHz, DMSO- *d*_6_): δ 8.28 (brs, 3H), 4.14 (t, *J* = 6.5 Hz, 2H), 2.95 (s, 2H), 2.82–2.76 (m, 4H), 2.02 (s, 3H). ^13^C NMR (75 MHz, DMSO-*d*_6_): δ 170.3, 62.9, 38.4, 29.4, 28.1, 20.7. HRMS (ESI): *m*/*z* [M + H]^+^ calcd for [C_6_H_13_O_2_NS + H]^+^, 164.0740; found, 164.0737.

### Continuous Flow Procedure for the Photochemical Thiol–Ene Reaction of Cysteamine Hydrochloride with VC (Table 1)

The liquid feed solution was prepared by dissolving cysteamine hydrochloride, 9-FL, and methyl benzoate as the internal standard in a volumetric flask (25 mL) in MeOH. The solution was degassed by sparging with Ar using a balloon and needle. The thermostats were set to the desired temperature beforehand (respective temperature for the reaction, 15 °C LED-cooling). The liquid feed was directly pumped from the volumetric flask using a syringe pump (Syrris-Asia) at maximum flow rate (2.5 mL/min) until the reactor was filled with the substrate solution. Then, the flow rate was reduced to the desired value, the LEDs were turned on (365 nm, 100% intensity), and the MFC was set to deliver the desired amount of VC. After reaching a steady state (about 20 min), the sample was collected. 100 μL of this sample was diluted with 500 μL of MeOH-*d*_4_ and analyzed by ^1^H NMR (300 MHz).

### Telescoped Continuous Flow Procedure for the Synthesis of Thiomorpholine

The liquid feed solution was prepared by dissolving cysteamine hydrochloride (45.44 g, 0.4 mol), 9-FL (0.36 g, 2 mmol, 0.5 mol %), and diphenyl ether (7.264 g, 0.04 mol) as the internal standard in a volumetric flask (100 mL) in MeOH. Dissolution was aided by sonication. The thermostats were set to the desired temperature beforehand (20 °C reaction, 15 °C LED-cooling). The liquid feed was directly pumped from the volumetric flask using a syringe pump (Syrris-Asia) at the maximum flow rate (2.5 mL/min) until the reactor was filled with the substrate solution. Then, the flow rate was reduced to the desired value (0.139 mL/min), the LEDs were turned on (365 nm, 100% intensity), and the MFC was set to deliver the desired amount of VC (12.1 mLn/min, p = 0.8–0.9 bar). After reaching a steady state (about 20 min), the output of the reactor was connected to the gas separator/hold vial. About 15 min later, the two pumps delivering the thiol–ene mixture and DIPEA (neat) were turned on and set to the corresponding flow rates (see Scheme 4). The sonication was turned on, and the ultrasonic bath was set to the desired temperature (80 °C) beforehand. The temperature of this water bath was equilibrated between 76 and 78 °C and was monitored by a K-type thermometer. 50 min later, the cyclization reaction had reached a steady state, and the reactor output was collected for 7 h (7 fractions of 1 h each). During this time, the thiol–ene mixture in the hold vial was sampled every 1 h [100 μL diluted with 500 μL of MeOH-*d*_4_ and analyzed by ^1^H NMR (300 MHz)]. The output of the cyclization reaction was collected every 30 min [100 μL diluted with 500 μL of MeOH-*d*_4_ and analyzed by ^1^H NMR (300 MHz)]. To the combined fractions, 1 M HCl (140 mL) and EtOAc (300 mL) were added. After separation of the phases, the organic phase was washed with 1 M HCl (3 × 25 mL) until no more thiomorpholine could be detected in the organic phase by LC–MS. Next, ∼4 M NaOH was added to the combined aq. phases until pH > 13 and extracted 3× with DCM. Additional NaOH was added because the pH dropped to ∼12. The aqueous phase was further extracted with DCM until no more thiomorpholine could be detected in the aqueous phase by LC–MS. The combined organic fractions were dried over Na_2_SO_4_, filtered, and the solvent was removed by evaporation (100 mbar at 40 °C water bath). After vacuum distillation, 12.74 g (54% overall) of thiomorpholine (**1**) was obtained as a colorless oil. Bp: 58–64 °C at 20 mbar. ^1^H NMR (300 MHz, CDCl_3_): δ 3.09–3.05 (m, 4H), 2.57–2.53 (m, 4H), 1.52 (brs, 1H). ^13^C NMR (75 MHz, CDCl_3_): δ 47.9, 28.3. The data are in agreement with previously published values.^6,8^

## Supporting Information Available

Additional experimental details, photographs of reactor setup, further optimization studies, and ^1^H NMR and ^13^C NMR spectra (PDF)
