# Supplementary material for: Synthesis of Thiomorpholine via a Telescoped Photochemical Thiol–Ene/Cyclization Sequence in Continuous Flow
Source: Org Process Res Dev. 2022 Aug 4;26(8):2532–9. doi: 10.1021/acs.oprd.2c00214 (PMC9396661; doi:10.1021/acs.oprd.2c00214)
Supplement: Supplementary file 1 — op2c00214_si_001.pdf [file op2c00214_si_001.pdf]

Supporting Information

Synthesis of Thiomorpholine via a Telescoped Photochemical Thiol-ene/Cyclization Sequence in Continuous Flow

Alexander Steiner,[a,b] Ryan C. Nelson,[c] Doris Dallinger,[a,b,*] and C. Oliver Kappe[a,b,*]

[a]Institute of Chemistry, University of Graz, NAWI Graz, Heinrichstrasse 28, 8010 Graz, Austria

[b]Center for Continuous Flow Synthesis and Processing (CCFLOW), Research Center Pharmaceutical Engineering GmbH (RCPE), Inffeldgasse 13, 8010 Graz, Austria

[c]Medicines for All Institute, Virginia Commonwealth University, 737 North Fifth Street, Box 980100, Richmond, Virginia 23298, United States

Table of Contents

1. Equipment S2
2. Routes toward Sutezolid S3
3. Thiol-ene Reaction of Cysteamine and Vinyl Acetate S4
4. Thiol-ene Reaction of Cysteamine Hydrochloride and Vinyl Acetate S6
   4.1 Product Identification S6
   4.2 Batch Thiol-ene Reaction S8
   4.3 Optimization of the Continuous Flow Photochemical Thiol-ene Reaction S9
5. Light Aborbance of Cysteamine Hydrochloride S10
6. Thiol-ene Reaction of Cysteamine Hydrochloride and Vinyl Chloride S11
   6.1 Condensation of VC S11
   6.2 Optimizations and Experimental Procedure in Batch S12
   6.3 Optimizations in Flow S13
   6.4 Isolation of Intermediate 4 .... S14
7. Cyclization to Thiomorpholine S16
   7.1 Base Screening in Batch S16
   7.2 Optimization of the Telescoped Continuous Flow Procedure toward Thiomorpholine S16
   7.3 Isolation of Thiomorpholine S17
8. Photographs of the Reactor Set-ups S18
9. NMR Spectra S20
10. References S21

1. Equipment

Vinyl chloride gas cylinder: from AirLiquide (UN1086) contained stabilized vinyl chloride 2.8 (99.8%). An adjustable pressure reducer 0.05−1 bar was employed.

Mass flow controller: calibrated for vinyl chloride from Bronkhorst with 0.5 bar inlet and 1 bar outlet pressure, max flow: 150 mLn/min

Gas detector: Dräger Pac 8000 portable detector. Calibrated with a bumpstation using 10 ppm C_2H_4O/N_2 as calibration gas mixture.

Connectors and tubing: fittings from IDEX Health & Science Technologies were used. High purity PFA (perfluoroalkoxy) tubing with either 0.8 mm ID/1.6 mm OD or 1.6 mm ID/3.2 mm OD was used.

Photoreactor: a Corning Advanced-Flow Lab Photo Reactor was used. The reactor module (G1LF fluidic module) consisted of a compact glass fluidic module (155 × 125 × 8 mm size, 0.4 mm channel depth, 2.77 mL internal volume), encased within a high-capacity heat exchange channel (20 mL volume). LED panels were mounted on both sides of the fluidic module (40 mm from the center of the process stream). Each LED panel was equipped with 20 LEDs of 6 different wavelengths (120 LEDs in total) and a heat exchanger (T = 15 °C). The LED wavelength and intensity was controlled externally using a web-based interface, connected wirelessly to a router. Thermal regulation of the LED panels was carried out using a Huber Minichiller 280 filled with 30% ethylene glycol in water. Thermal regulation of the glass fluidic module was carried out using a Huber Ministat 230 filled with silicon oil (–20 °C to 195 °C).

2. Routes toward Sutezolid

a) Synthesis via a Modified Linezolid Route[S1]

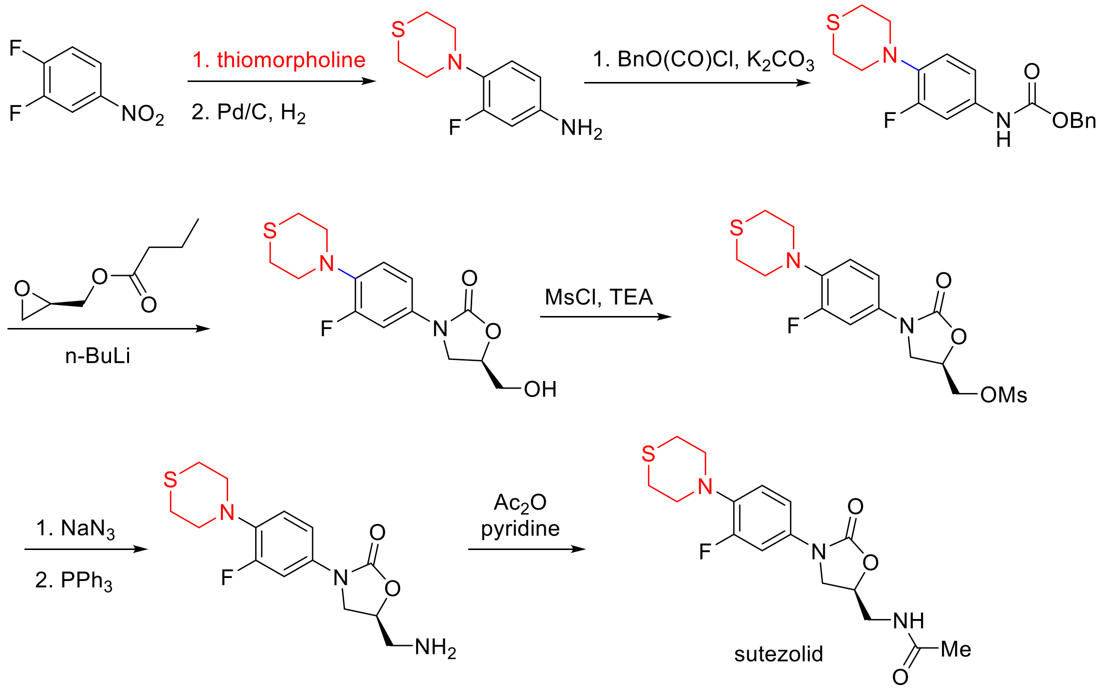

b) Convergent Approach Toward Sutezolid[S2]

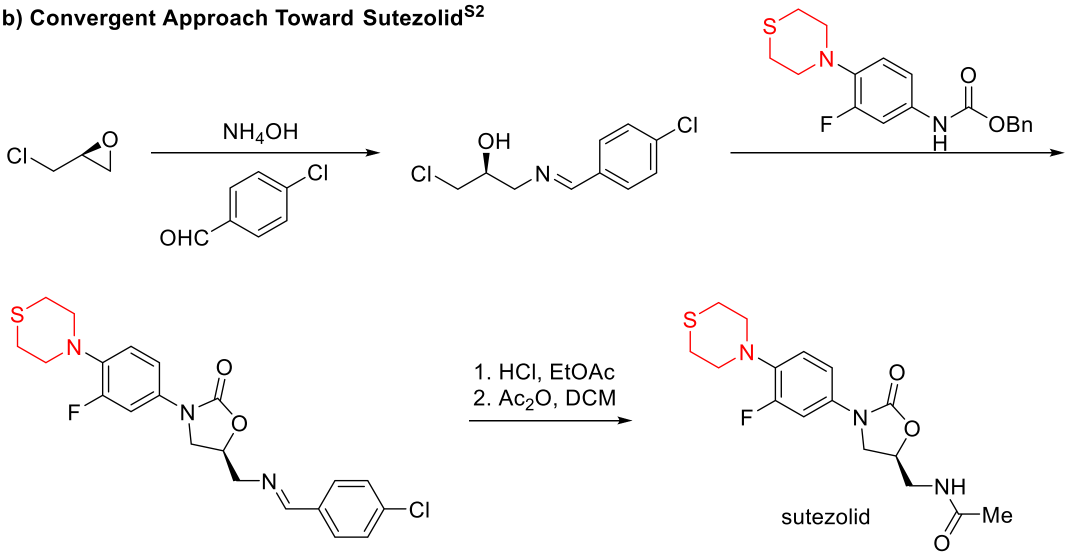

Scheme S1. Reported routes toward sutezolid.

3. Thiol-ene Reaction of Cysteamine and Vinyl Acetate

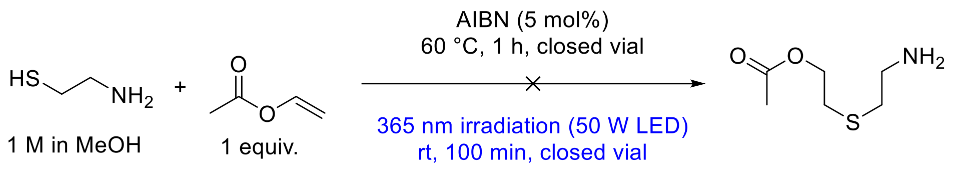

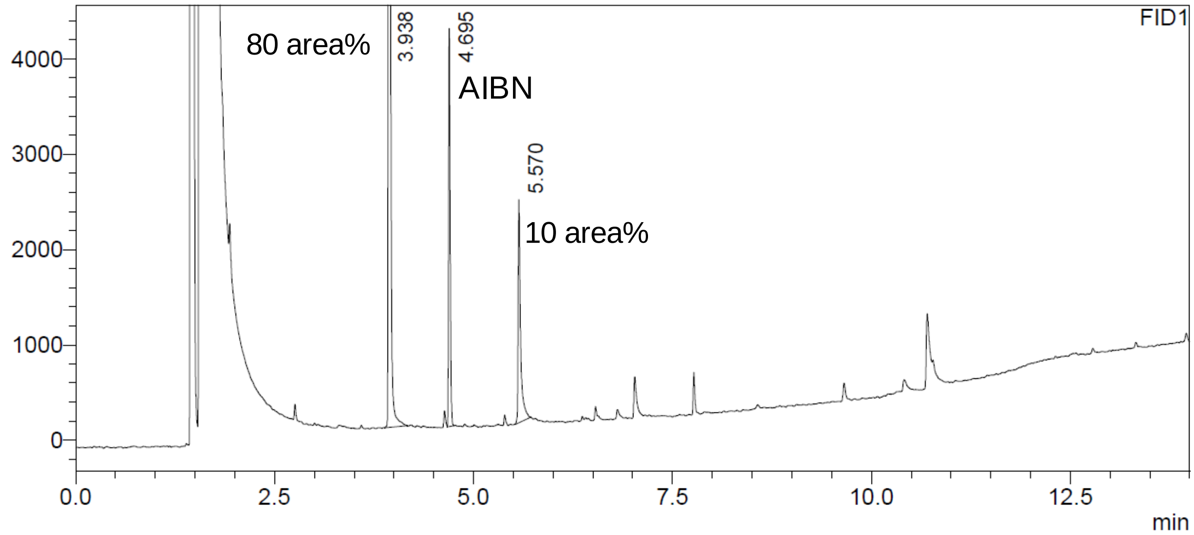

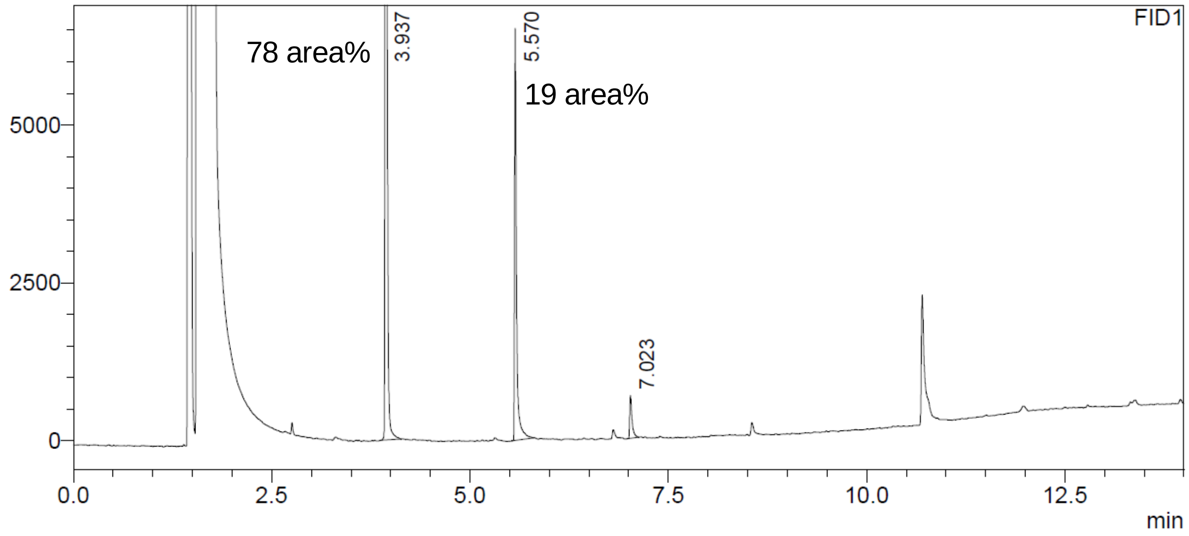

Figure S1. Thiol-ene reaction of cysteamine and vinyl acetate in batch. GC-FID spectra of the crude reaction mixture. Top: thermal reaction. Bottom: photochemical reaction.

General batch procedure thermal: A 4 mL screw-cap vial was charged with cysteamine (77.2 mg, 1 mmol), vinyl acetate (1 equiv.), AIBN as initiator (5 mol%), and 1 mL of MeOH was added. The vial was closed using high p/T caps and heated in an Al-block to 60 °C.

General batch procedure photochemical: A 5 mL conical microwave vial was charged with cysteamine (77.2 mg, 1 mmol) and vinyl acetate (1 equiv.), and 1 mL of MeOH was added. The solution was sparged with Ar for ~1min to remove oxygen. The vial was closed using a crimp cap and irradiated using a 365 nm, 50 W LED lamp (7 cm distance to center of vial).

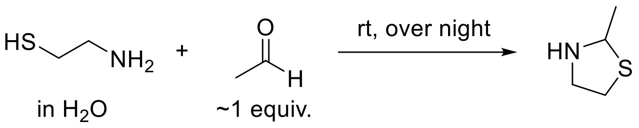

a)

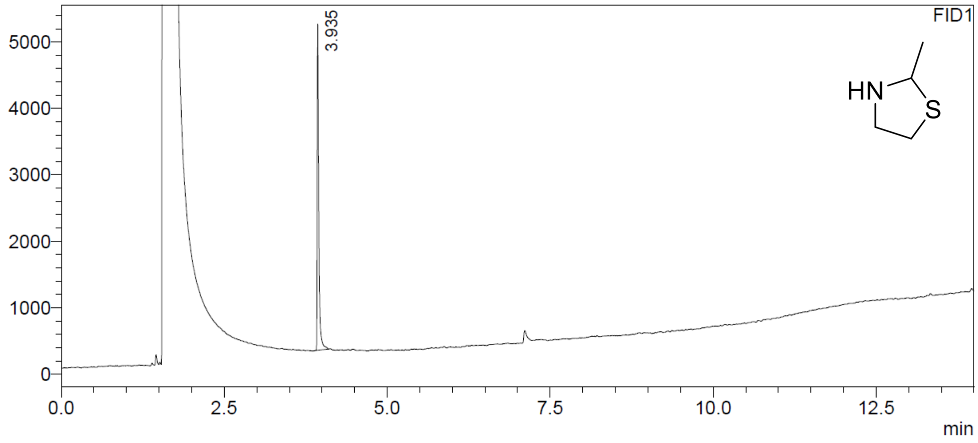

b)

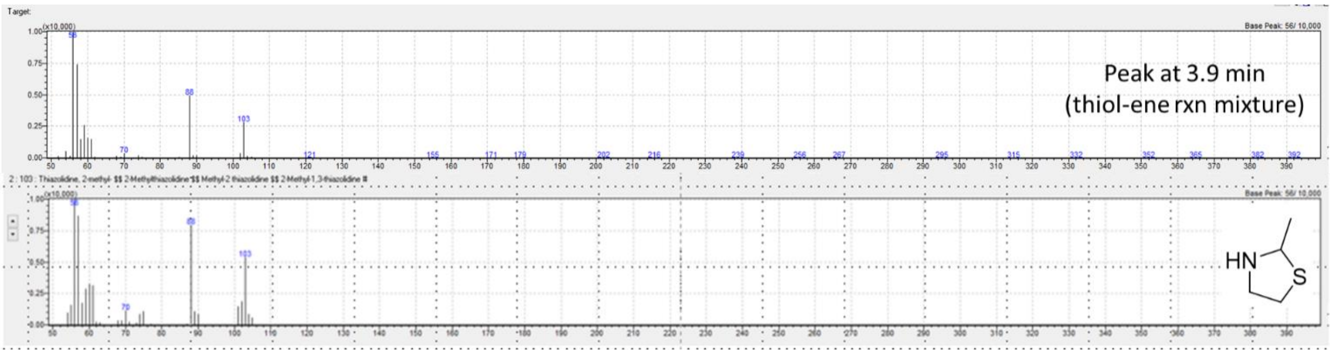

Figure S2. Product identification of the batch thermal and photochemical thiol-ene reaction of cysteamine and vinyl acetate. The synthesis of 2-methyl-1,3-thiazolidine was performed according to Ref S3. a) GC-FID spectra of isolated 2-methyl-1,3-thiazolidine. b) Mass fragmentation (GC-MS) of the peak at 3.94 min. Top: thiol-ene reaction mixture. Bottom: suggested structure from the NIST17R mass spectral library (included in the software).

4. Thiol-ene Reaction of Cysteamine Hydrochloride and Vinyl Acetate

4.1 Product Identification

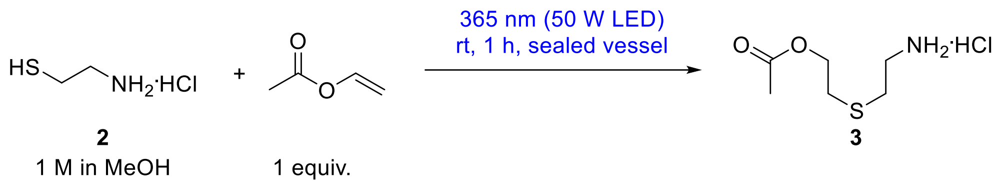

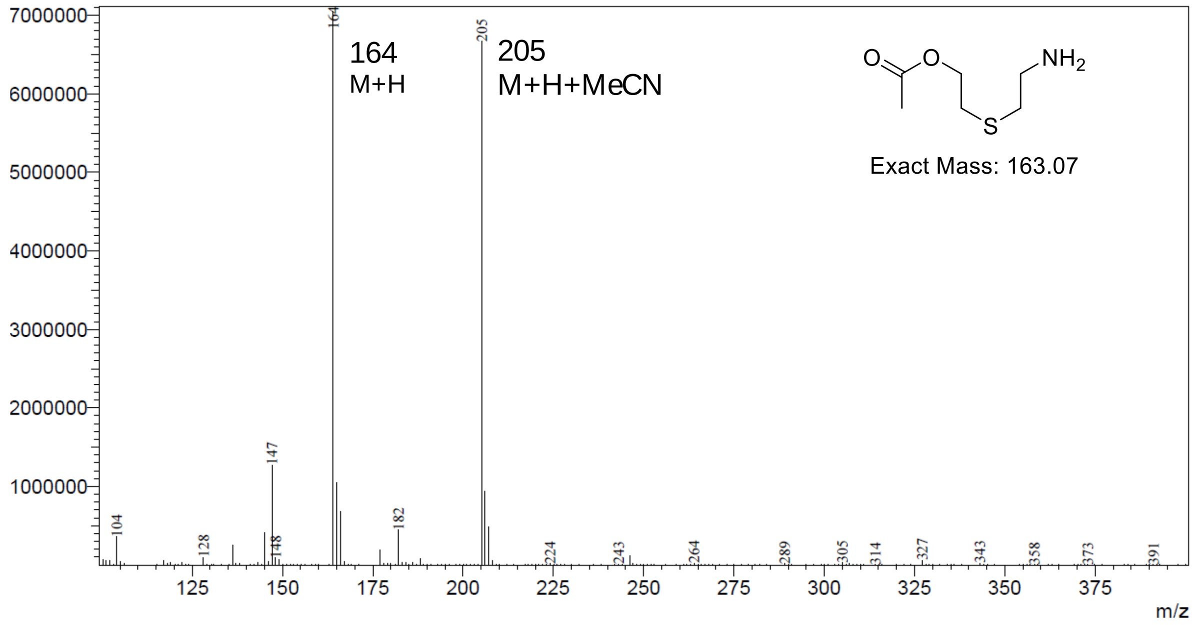

Figure S3. Product idendification of the batch photochemical thiol-ene reaction of cysteamine hydrochloride and vinyl acetate. LC-MS analysis of the isolated product.

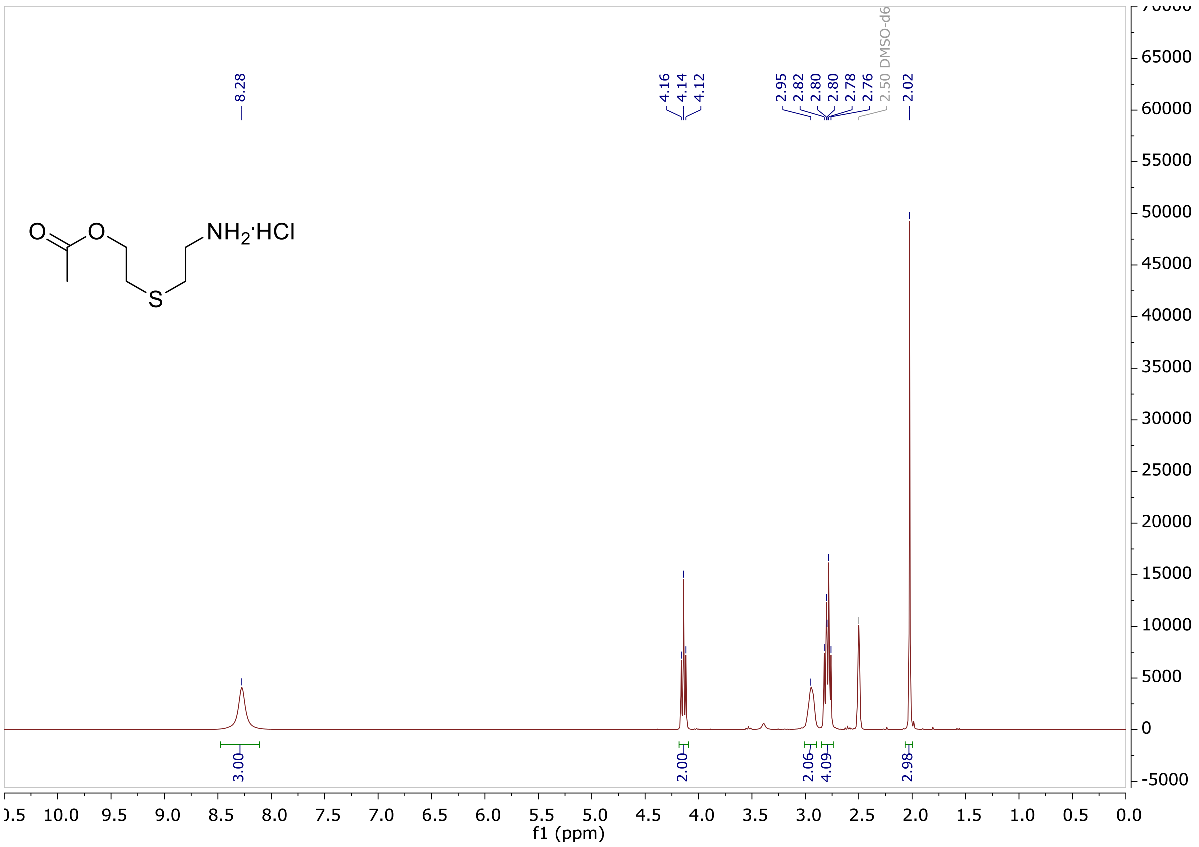

Figure S4. ^{1}H NMR (DMSO-d_6, 300 MHz) of the isolated product of of the batch photochemical thiol-ene reaction of cysteamine hydrochloride with vinyl acetate.

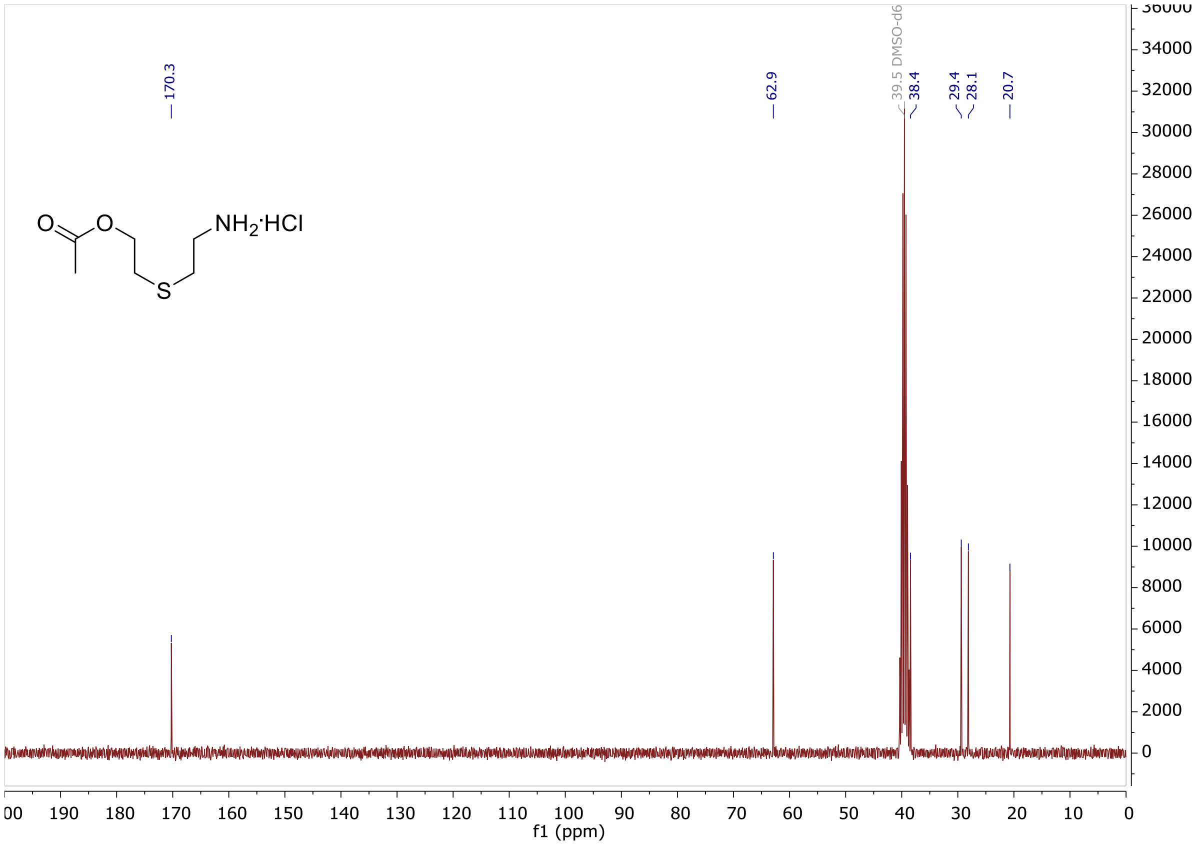

Figure S5. ^{13}C NMR (DMSO-d_6, 75 MHz) of the isolated product of of the batch photochemical thiol-ene reaction of cysteamine hydrochloride with vinyl acetate.

4.2 Batch Thiol-ene Reaction

The photochemical route not only provided full conversion, but also a very clean reaction profile compared to the thermal reaction (Figure S6).

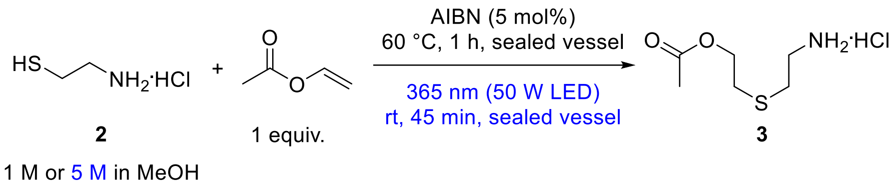

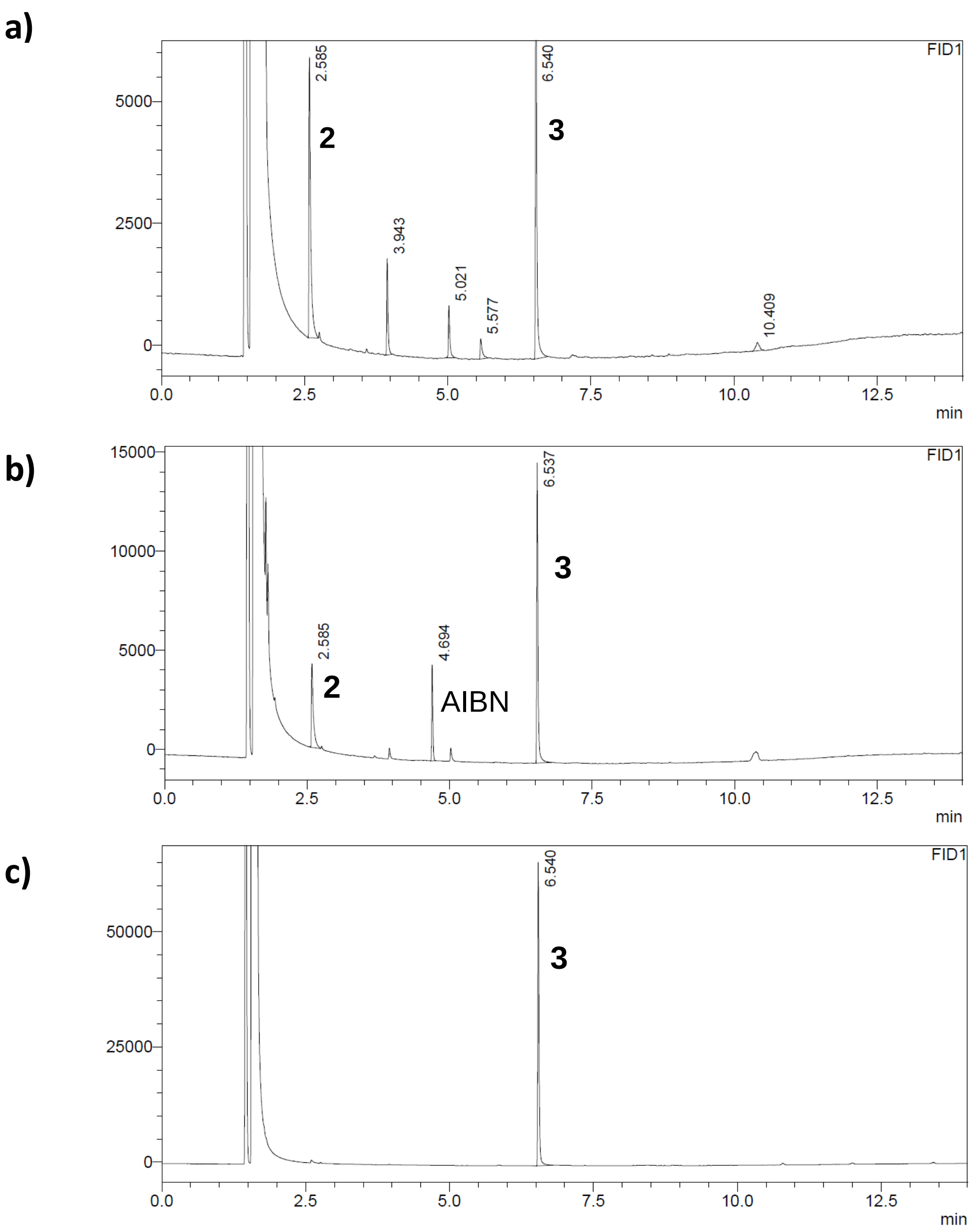

Figure S6. GC-FIDs of the batch thiol-ene reaction of cysteamine hydrochloride and vinyl acetate. a) Crude reaction mixture: thermal. b) Crude reaction mixture: thermal with 5 mol% AIBN. c) Crude reaction mixture: photochemical.

4.3 Optimization of the Continuous Flow Photochemical Thiol-ene Reaction

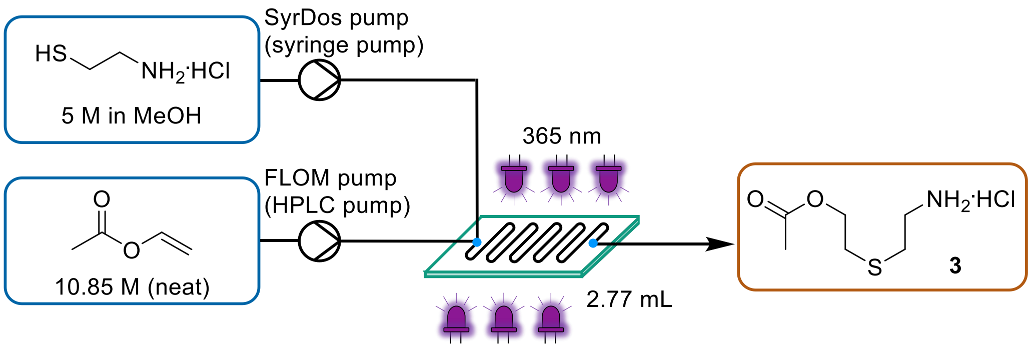

Figure S7. Reactor set-up: The Corning Advanced-Flow photoreactor and LED irradiation at 365 nm was used.

For the optimization, a temperature and residence time screening was performed. A 5 M solution of cysteamine hydrochloride was prepared by dissolving 14.2 g in MeOH (25 mL). The thermostats were set to the desired temperatures (respective temperature for the reaction, 15 °C LED cooling) and the LEDs were turned on at the desired wavelength. The pumps were set to the desired flow rates. If not otherwise stated in Figure S8, 1.1 equiv of vinyl acetate are used. After allowing the reactor to reach steady state, the reactor output was collected for exactly 1 min into a flask containing biphenyl as external standard and then analyzed by GC-FID. However, clogging issues were encountered during the thiol-ene reaction at such high concentration.

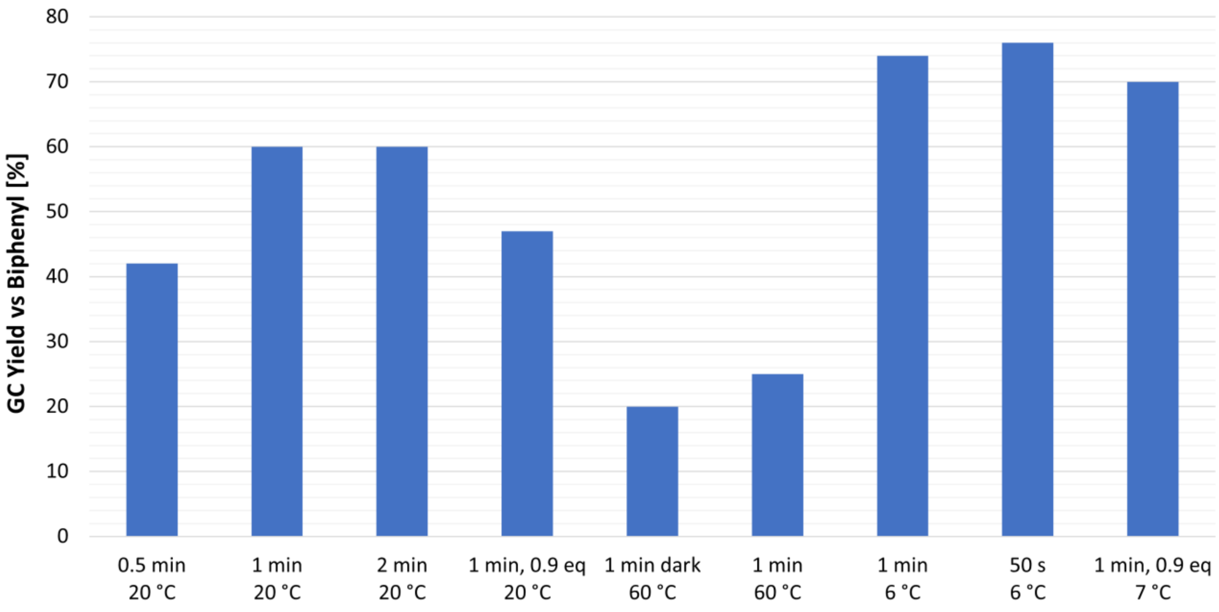

Figure S8. Optimization of the photochemical thiol-ene reaction in flow.

5. Light Aborbance of Cysteamine Hydrochloride

UV-vis spectroscopy revealed an extremely low absorption coefficient at the irradiation wavelength of the LED (ε = 0.025 $Lmol^{-1}cm^{-1}$ at 363 nm). This would indicate poorer performance in flow, since at a concentration of 1 M of **2** only about 1% of the incident light is absorbed due to the shorter path length of 0.04 cm (plate´s channel size), compared with about 7% in batch with a path length of 1.3 cm (diameter of the vial). At higher concentrations, also the light absorbance increases accordingly.

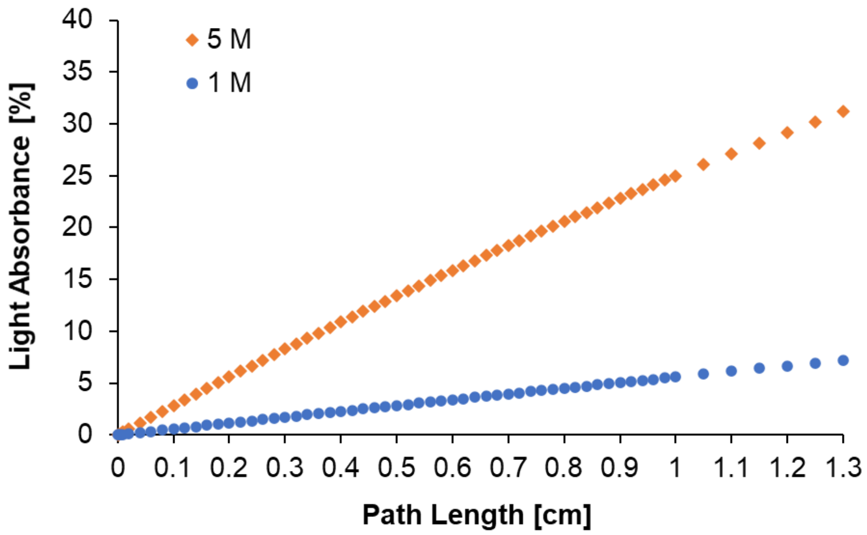

Figure S9. Light absorbance as a function of path length utilizing the Beer−Lambert law of **2** at different concentrations.

6. Thiol-ene Reaction of Cysteamine Hydrochloride and Vinyl Chloride

6.1 Condensation of VC

A 5 mL conical microwave vial equipped with a magnetic stir bar was charged with 2 mL of a solution containing cysteamine hydrochloride (1 M) and internal standard (methyl benzoate) in MeOH (HPLC grade). For the thermal reaction, AIBN (16 mg, 5 mol%, 0.1 mmol) was additionally added. The vial was closed (cap with septum), weighed (tara), immersed in an acetone bath and cooled with liquid N_2 below −50 °C (usually ~−60 °C). Under vigorous stirring of the solution, VC was charged (10 mLn/min) via a cannula that was immersed into the cold solution, whilst a second cannula prevents buildup of pressure (open to fume hood). After charging of the VC was finished (confirmed by weighing the vial), all cannulas were removed and the solution was allowed to warm to rt.

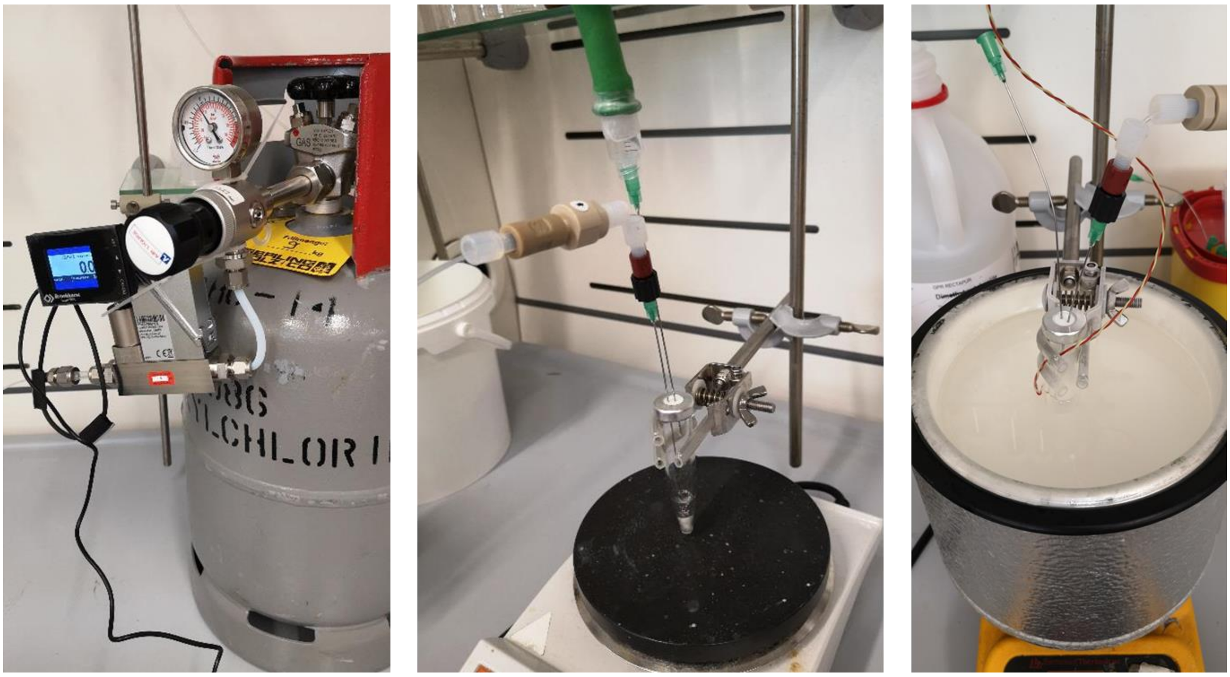

Figure S10. Set-up for condensing VC into a solution of cysteamine hydrochloride in MeOH. A Bronkhorst mass flow controller was used to accurately dose VC.

6.2 Optimizations and Experimental Procedure in Batch

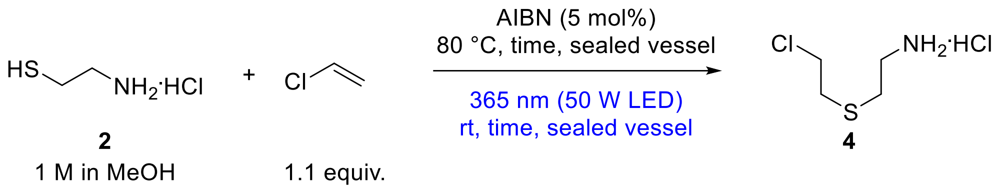

	Time [min]	AIBN [mol%]	NMR yield [%]
Thermal	30	5	83
	10	5	78
	2	5	68
	2	–	no conversion of cysteamine
Photochemical	30	–	>99

Thermal: After condensation of VC as described in 6.1., the reaction mixture was then heated at 80 °C in the microwave reactor for the indicated time. After removal of remaining VC by sparging with Ar, 100 µL of the reaction mixture were mixed with 500 µL of MeOH-d4 and analyzed by ^{1}H NMR.

Photochemical: After condensation of VC as described in 6.1., the reaction mixture was then irradiated (50 W LED, 365 nm, 7 cm distance to center of vial, see Figure S14) for the desired time. After removal of remaining VC by sparging with Ar, 100 µL of the reaction mixture were mixed with 500 µL of MeOH-d4 and analyzed by ^{1}H NMR.

6.3 Optimizations in Flow

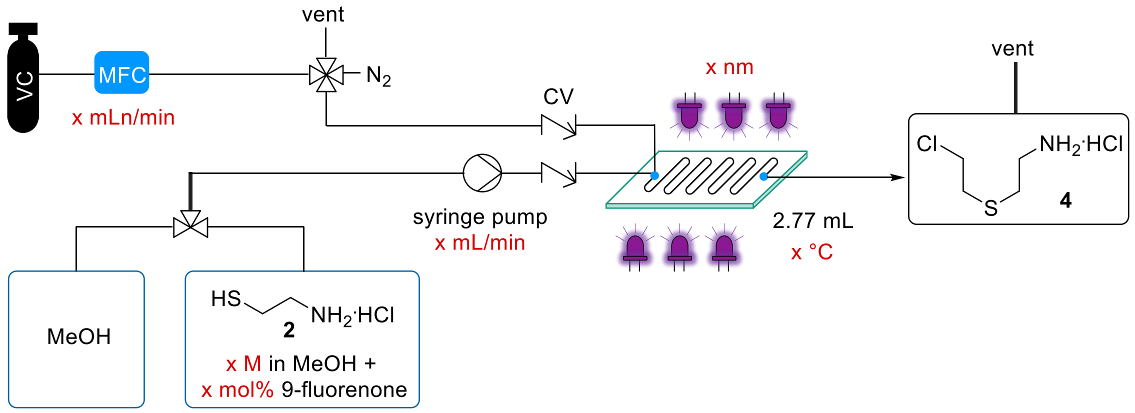

Table S1. Optimization studies of the continuous thiol-ene reaction of cysteamine hydrochloride (**2**) and vinyl chloride (VC).

Entry	Conc of 2 [M]	9-FL [mol%]	T [°C]	Equiv [VC]	Wavelenght [nm]	Flow rates [mL/min]		NMR yield [%]
						2	VC	
1	1	–	20	1.1	365	0.277	6.6	53
2	1	–	20	1.0	365	0.277	6.1	58
3	1	–	6	1.0	365	0.277	6.1	18
4[a]	1	5	20	1.0	365	0.277	6.1	87
5	1	5	20	1.0	405	0.277	6.1	89
6	1	5	6	1.0	365	0.277	6.1	83
7[a]	2	5	20	1.0	365	0.277	12.1	93
8	2	5	20	1.0	405	0.277	12.1	91
9	2	5	6	1.0	365	0.277	12.1	91
10[b]	4	5	20	1.0	365	0.139	12.1	>99
11	4	1	20	1.0	365	0.139	12.1	>99
12	4	0.1	20	1.0	365	0.139	12.1	>99
13	4	–	20	1.0	365	0.139	12.1	98
14	4	5	20	1.0	405	0.139	12.1	>99
15	4	1	20	1.0	405	0.139	12.1	>99
16	4	0.1	20	1.0	405	0.139	12.1	90
17	4	–	20	1.0	405	0.139	12.1	54
18	4	5	20	1.0	475	0.139	12.1	47

a...Degassing of the liquid substrate feed with Ar.
b...With or without degassing.

The liquid feed solution was prepared by dissolving cysteamine-hydrochloride, 9-fluorenone, and methyl benzoate as internal standard in a volumetric flask (25 mL) in MeOH. Where indicated in Table S1, the solution was degassed by sparging with Ar using a balloon and needle. The thermostats were set to the desired temperature beforehand (respective temperature for the reaction, 15 °C LED-cooling). The liquid feed was directly pumped from the volumetric flask using a syringe pump (Syrris-Asia) at maximum flow rate (2.5 mL/min) until the reactor was filled with the substrate solution. Then, the flow rate was reduced to the desired value, the LEDs were turned on (100% intensity) and the MFC was set to deliver the desired amount of VC. After reaching steady-state (about 20 minutes), a sample was collected. 100 µL of this sample were diluted with 500 µL of MeOH-d4 and analyzed by ^{1}H NMR (300 MHz).

6.4 Isolation of Intermediate 4

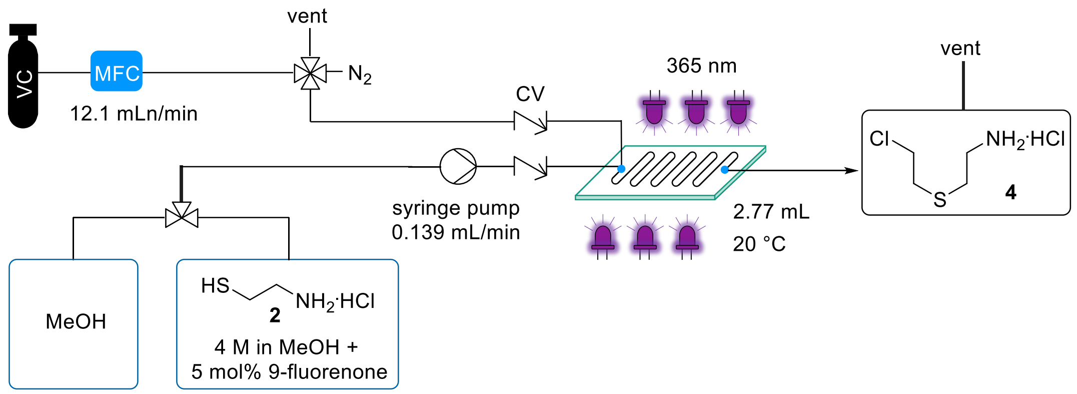

The liquid feed solution was prepared by dissolving cysteamine-hydrochloride (11.36 g, 0.1 mol) and 9-fluorenone (0.90 g, 5 mmol, 5 mol%) in a volumetric flask (25 mL) in MeOH. The solution was degassed by sparging with Ar using a balloon and needle. The thermostats were set to the desired temperature beforehand (20 °C reaction, 15 °C LED-cooling). The liquid feed was directly pumped from the volumetric flask using a syringe pump (Syrris-Asia) at maximum flow rate (2.5 mL/min) until the reactor was filled with the substrate solution. Then, the flow rate was was reduced to the desired value (0.139 mL/min), the LEDs were turned on (365 nm, 100% intensity) and the MFC was set to deliver the desired amount of VC (12.1 mLn/min, p = 0.8–0.9 bar). After reaching steady-state (about 20 minutes) the product stream was collected for 12 min. 1.30 g (105%) of product was isolated after evaporation of the solvent as a mixture of 95.6% of **4** and 4.4% of 9-fluorenone according to ^{1}H NMR.

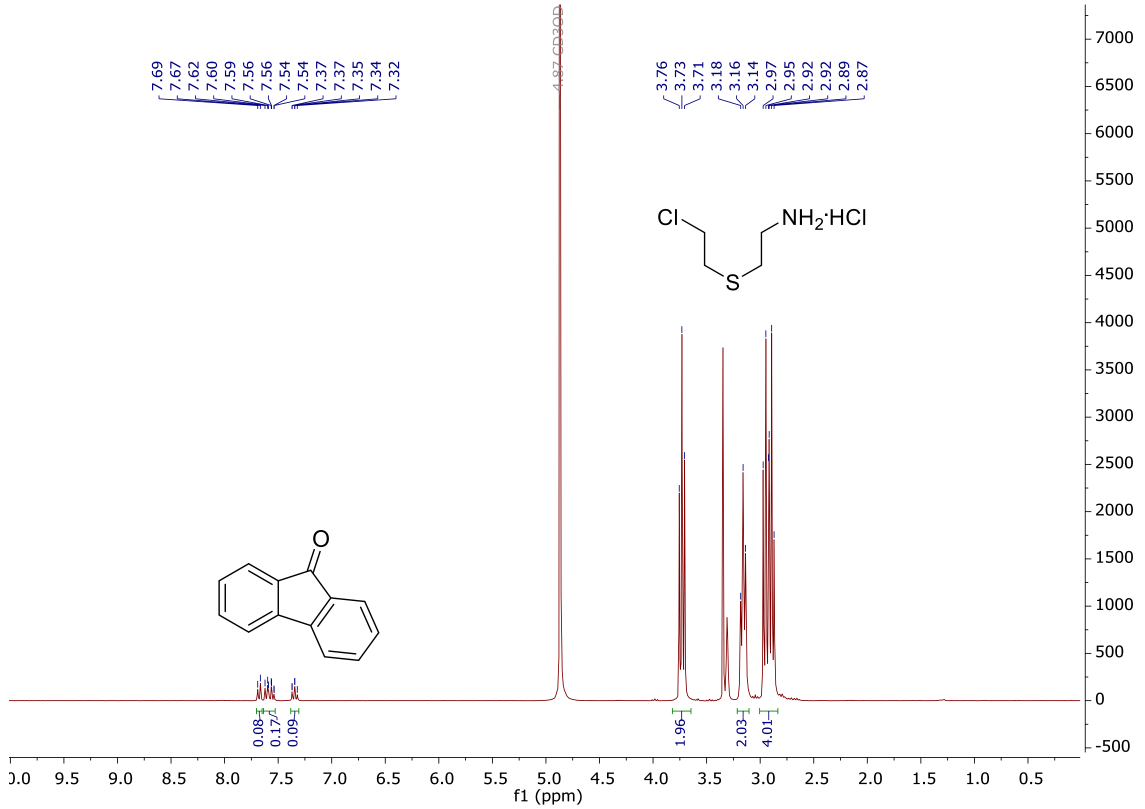

Figure S11. ^{1}H NMR (MeOH-d4, 300 MHz) of the isolated product of the flow photochemical thiol-ene reaction of cysteamine hydrochloride with vinyl chloride.

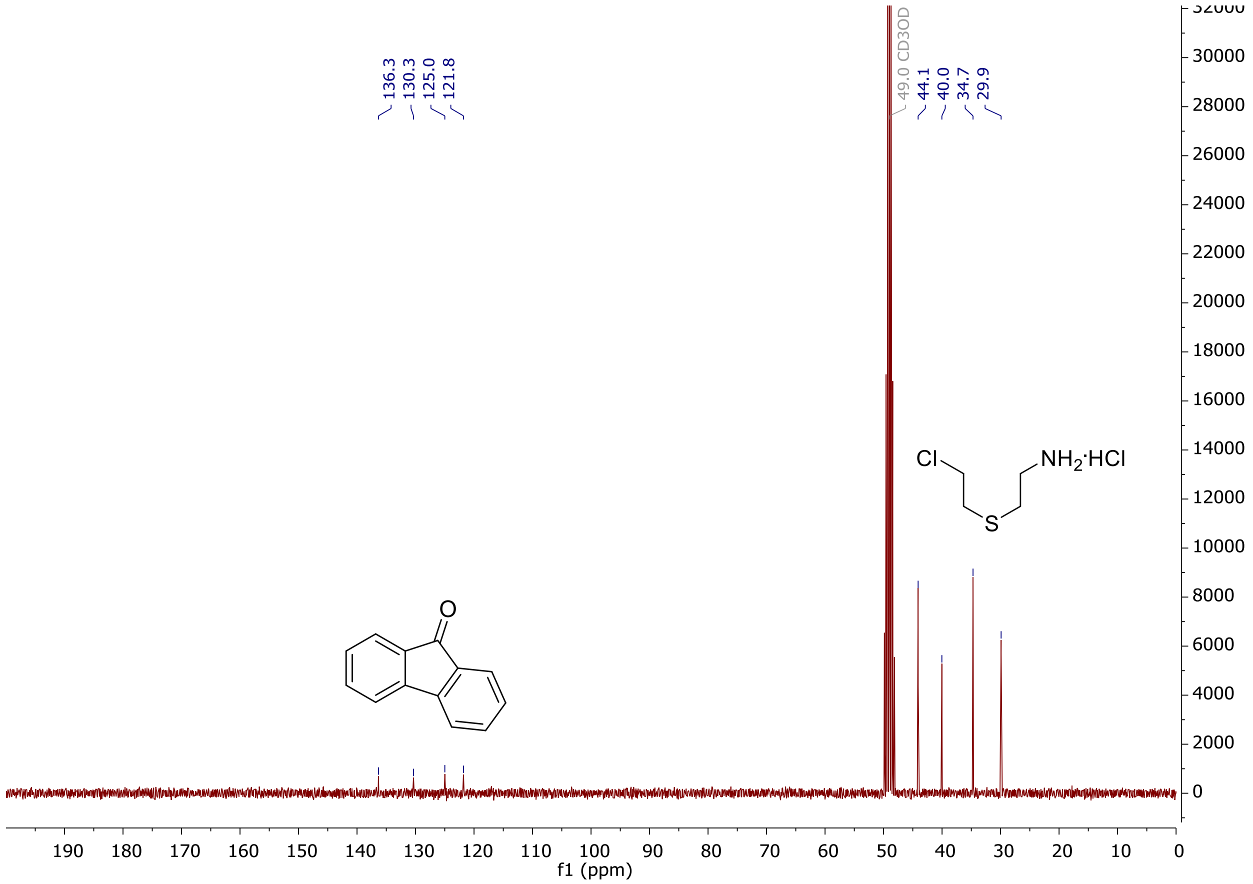

Figure S12. ^{13}C NMR (MeOH-d4, 75 MHz) of the isolated product of the flow photochemical thiol-ene reaction of cysteamine hydrochloride with vinyl chloride.

7. Cyclization to Thiomorpholine

7.1 Base Screening in Batch

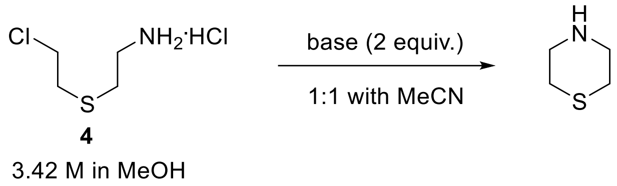

Base	^{1}H NMR yield (conv) thiomorpholine [%]		Appearance
	rt, 10 min	MW, 100 °C, 5 min	
Et_3N	12 (15)	86 (>99)	precipitation
Bu_3N	8 (10)	45 (47)	biphasic liquid
DIPEA	12 (15)	89 (>99)	monophasic
DBU	20 (23)	87 (>99)	monophasic

Table S2. Base screening for the cyclization of intermediate **3** to thiomorpholine. The crude solution of the thiol-ene reaction (3.42 M) was reacted with 2 equiv of base. Diphenyl ether was used as internal standard. MW: sealed vessel microwave heating

7.2 Optimization of the Telescoped Continuous Flow Procedure toward Thiomorpholine

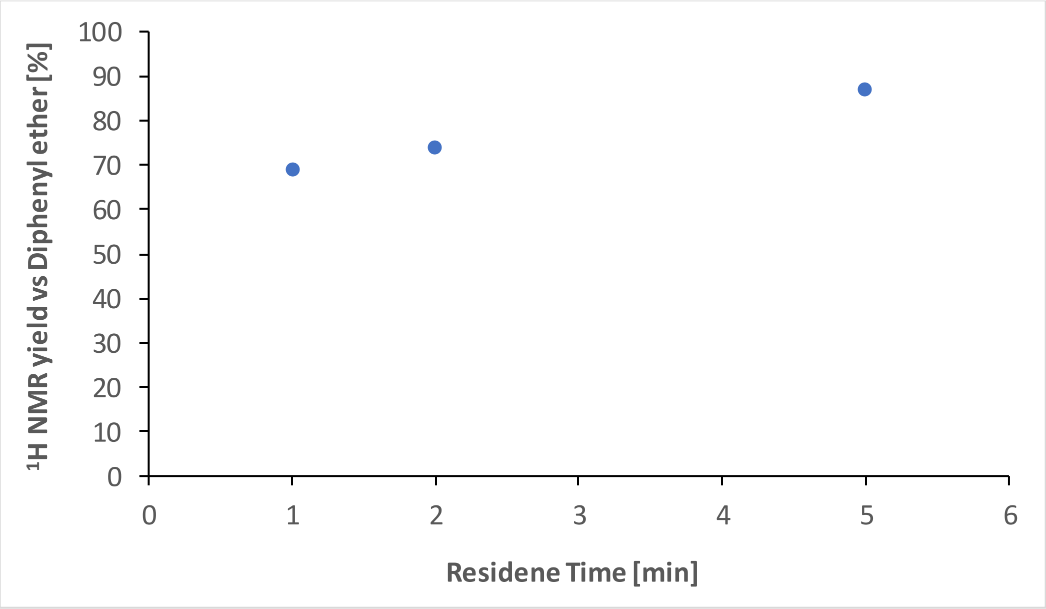

Figure S13. Optimization of the telescoped thiol-ene/cyclization reaction at 100 °C using a PFA coil (1.6 mm ID, 3.2 mm OD) filled with glass beads (0.4–0.6mm) providing 0.5 mL void volume when filled, as both mixing and reaction unit.

7.3 Isolation of Thiomorpholine

The fractions of the long run were combined and extracted as described in the Experimental Section. The organic phase was then evaporated and distilled under vacuum at 20 mbar.

Fraction	Bp [°C]	Mass [g]	Comment	Yield thiomorpholine [%]
1	25−26	2.28	DIPEA + Thiomorpholine	1 (NMR)
2	25−26	16.85	DIPEA + Thiomorpholine	7 (NMR)
3	58−64	11.91	Thiomorpholine	50 (isol)
residue	−	10.10		

Fractions 1 + 2 were combined and redistilled. An additional 4% of pure thiomorpholine was isolated, giving a total isolated yield of thiomorpholine of 54%. In the residue, peaks that could be assigned to thiomorpholine were detected in the NMR, but were not quantified. In addition, unidentified peaks were present. Possibly, thiomorpholine could either decompose under harsher conditions or further react with other unidentified side products under those conditions. The work-up and distillation procedures were performed only once for the long run experiment, and thus are not optimized.

8. Photographs of the Reactor Set-ups

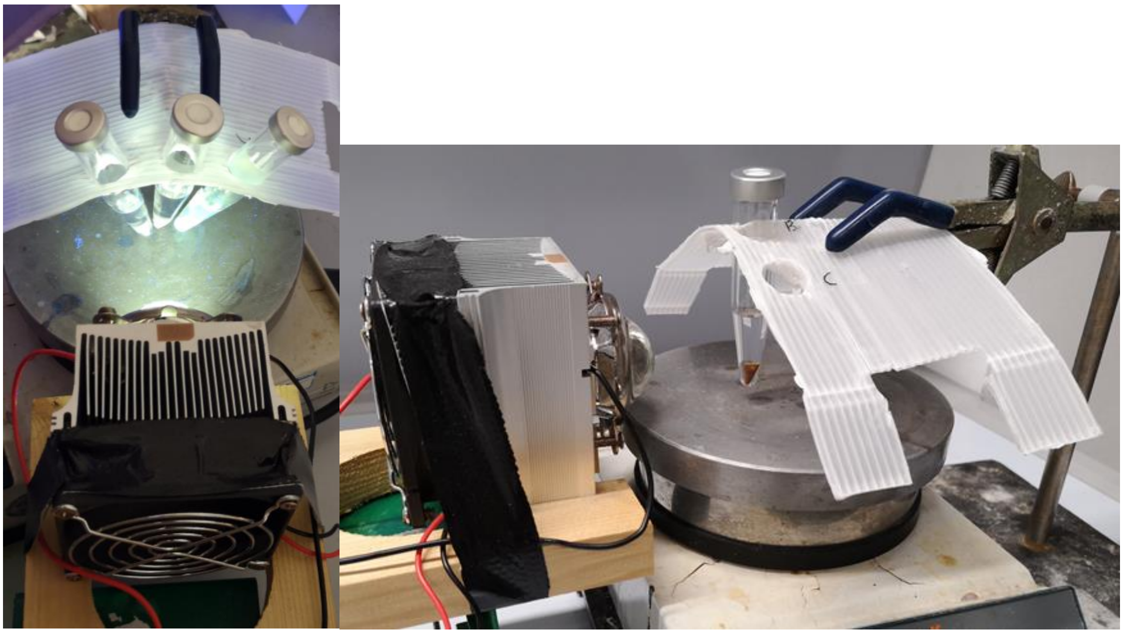

Figure S14. Set-up for batch photochemical thiol-ene reactions. A 365 nm, 50 W LED lamp was used, mounted at a distance of 7 cm to the vials.

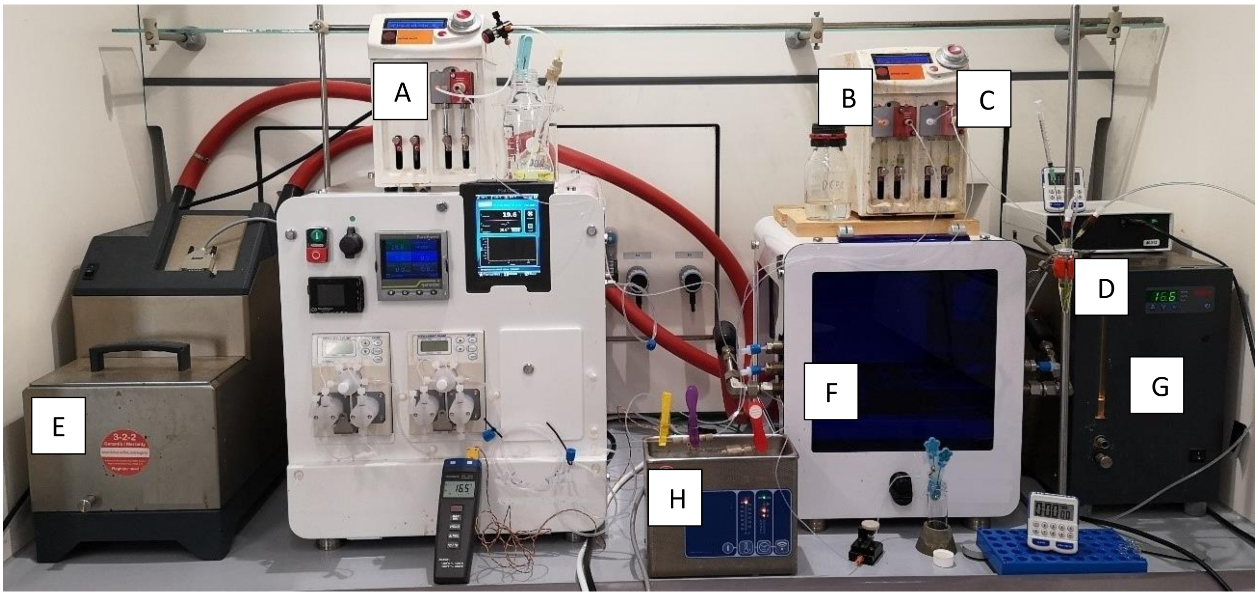

Figure S15. Set-up for the telescoped synthesis. The VC dosing unit is located in the adjacent fume hood. VC is fed to the reactor using PFA tubing (1.6 mm OD, 0.8 mm ID). A) Feed of cysteamine hydrochloride, 9-fluorenone and methyl benzoate (IS) in MeOH. B) DIPEA feed. C) Feed of thiol-ene mixture from photoreaction. D) Hold vessel/gas separator after photoreactor. E) Temperature control for reaction plate. F) Fluidic module housing, with tinted plastic panels for light containment. G) Temperature control for LED panels and wireless reciever for LED control. H) Ultrasonic bath with the submerged reaction coil for the cyclization reaction.

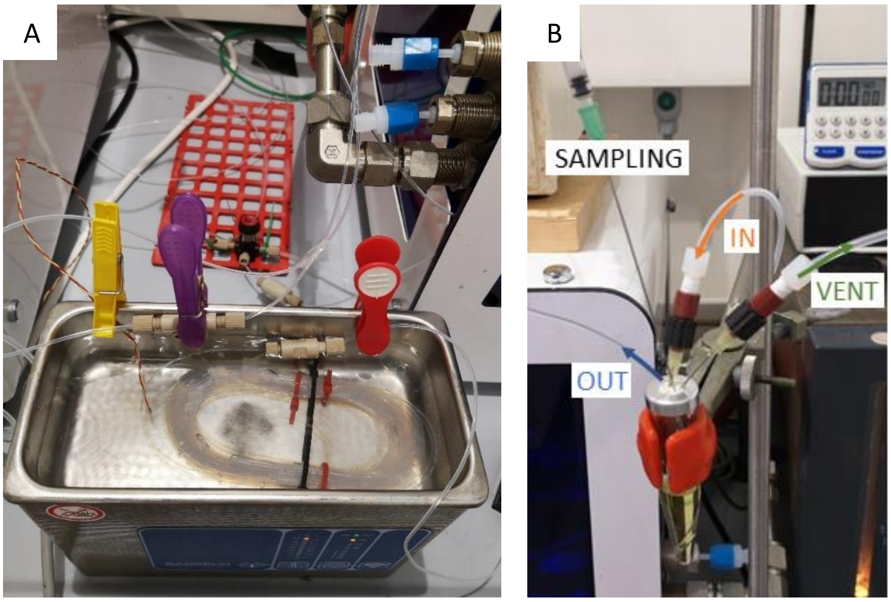

Figure S16. A) Close-up of the reaction coil submerged in water in an ultrasonic bath used for the cyclization reaction. B) Close-up of the hold vessel/gas separator.

9. NMR Spectra

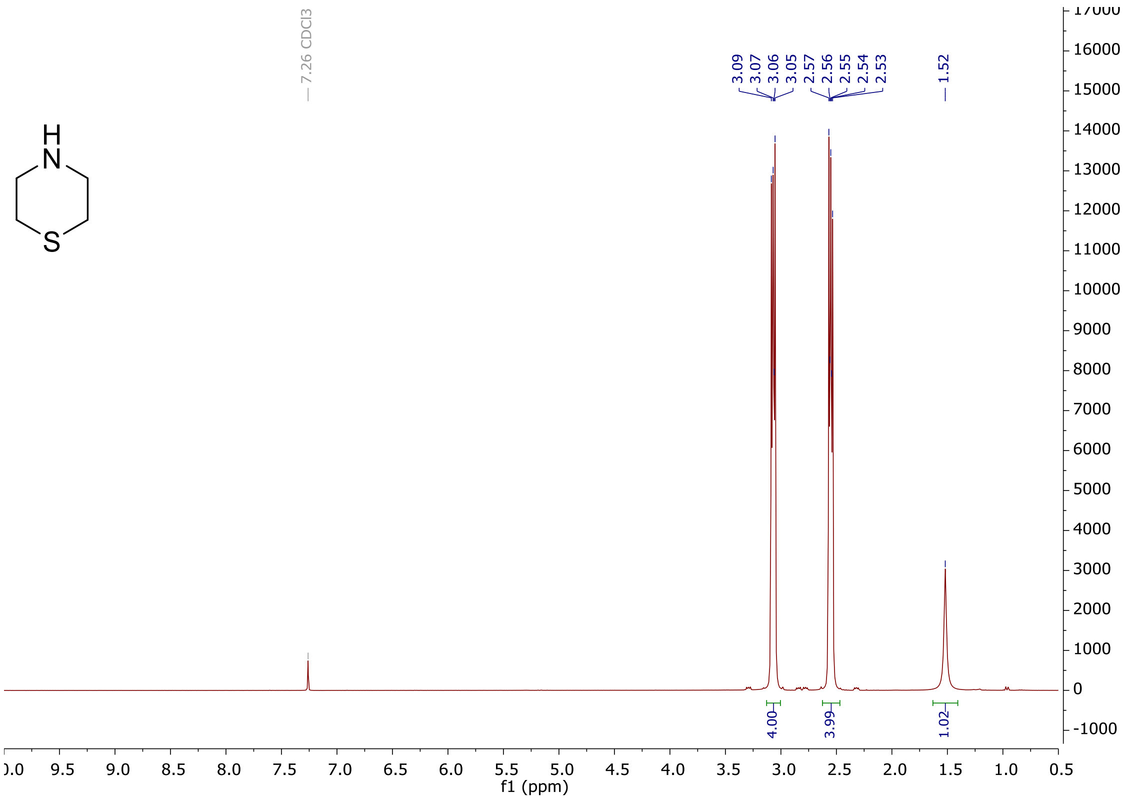

Figure S17. ^{1}H NMR ($CDCl_3$, 300 MHz) of isolated thiomorpholine of the telescoped thiomorpholine synthesis.

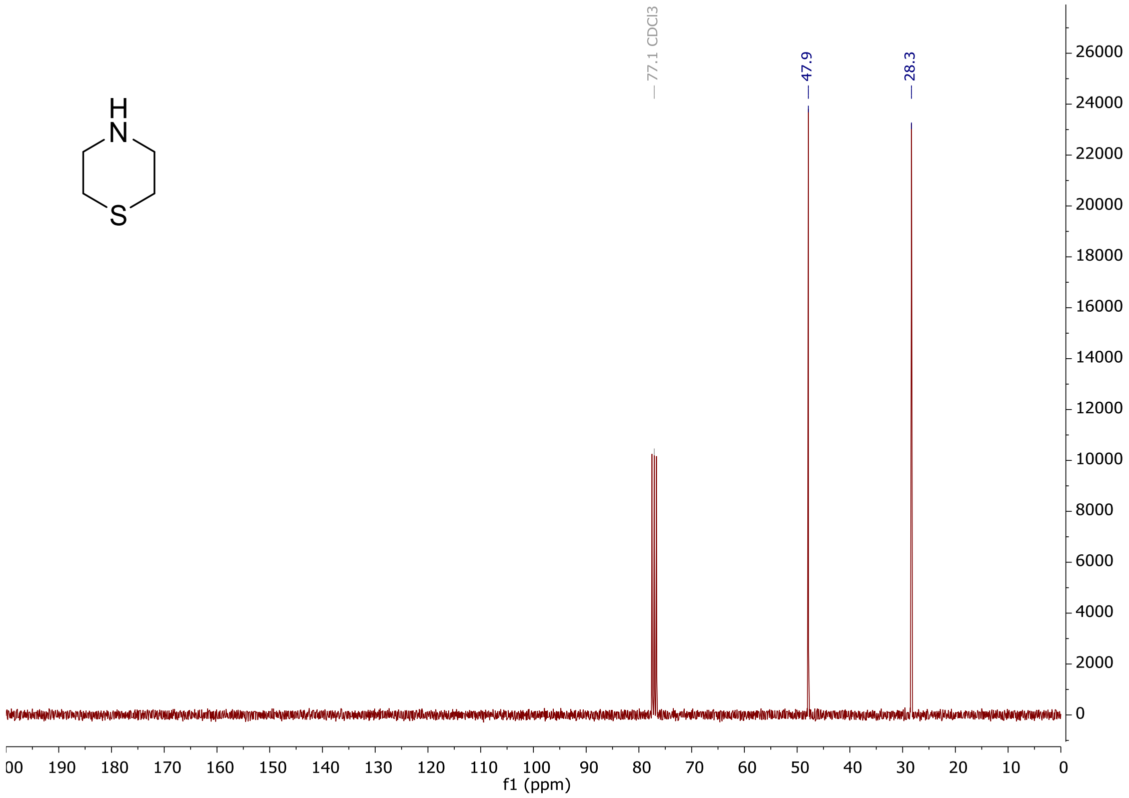

Figure S18. ^{13}C NMR ($CDCl_3$, 75 MHz) of isolated thiomorpholine of the telescoped thiomorpholine synthesis.

10.References

(S1) Arora, S. K.; Patil, V. J.; Nair, P. S.; Dixit, P. P.; Ajay, S.; Sinha, R. K. Antimycobacterial Compounds. US7691889B2, April 6, **2010**.

(S2) Pearlman, B. A.; Perrault, W. R.; Barbachyn, M. R.; Manninen, P. R.; Toops, D. S.; Houser, D. J.; Fleck, T. J. Process to Prepare Oxazolidinones. US5837870A, November 17, **1998**.

(S3) Yashurara A.; Shibamoto T. Determination of Physical and Spectral Data on Thiazolidines for Trace Aldehyde Analysis. *Agric. Biol. Chem*. **1989**, *53*, 2273−2274.
